# Quantifying CO_2_ forcing effects on lightning, wildfires, and climate interactions

**DOI:** 10.1126/sciadv.adt5088

**Published:** 2025-02-12

**Authors:** Vincent Verjans, Christian L. E. Franzke, Sun-Seon Lee, In-Won Kim, Simone Tilmes, David M. Lawrence, Francis Vitt, Fang Li

**Affiliations:** ^1^ Barcelona Supercomputing Center, Barcelona, Spain.; ^2^ Center for Climate Physics, Institute for Basic Science, Busan, Republic of Korea.; ^3^ Pusan National University, Busan, Republic of Korea.; ^4^ National Center for Atmospheric Research, Boulder, CO, USA.; ^5^ International Center for Climate and Environment Sciences, Institute of Atmospheric Physics, Chinese Academy of Sciences, Beijing, China.

## Abstract

Climate change affects lightning frequency and wildfire intensity globally. To date, model limitations have prevented quantifying climate-lightning-wildfire interactions comprehensively. We exploit advances in Earth System modeling to examine these three-way interactions and their sensitivities to idealized CO_2_ forcing in 140-year simulations. Lightning sensitivity to global temperature change (+1.6 ± 0.1% per kelvin) is mitigated by compensating atmospheric effects. Global burned area sensitivity to temperature (+13.8 ± 0.3% per kelvin) is largely driven by intensified fire weather and increased biomass but marginally by lightning changes. We find a universal law characterizing regional-scale modeled fire activity and its CO_2_ sensitivity, consistent with basic principles of statistical mechanics. Last, a negative climate feedback through intensified aerosol direct effect from fire emissions reaches an equivalent decrease of 0.91 ± 0.01% in CO_2_ radiative forcing. However, this feedback contributes to polar amplification. Our analysis shows that climate-lightning-wildfire interactions involve multiple compensating and amplifying feedbacks, which are sensitive to anthropogenic CO_2_ forcing.

## Teaser

A model analysis shows interactions between climate, lightning, wildfires, and changes in the interactions with global warming.

## INTRODUCTION

Lightning is the predominant cause of natural wildfire ignitions (*1*). Compared to anthropogenic fires, lightning-ignited wildfires (LIWs) show distinctive characteristics: They occur in more remote places, tend to burn larger areas, and are more sensitive to weather conditions (*2*). As a consequence, some ecosystems are particularly sensitive to LIWs, such as boreal and intact forests (*1*, *3*). More generally, natural wildfires are regulated by three climate-related factors: ignitions, fuel availability, and fire weather (*4*, *5*). While lightning affects the first factor, fuel availability refers to the biomass load, and fire weather refers to hot, dry, and windy weather promoting fire spread. This simple decomposition in three factors shows the complex interplay between wildfire and climate on a range of time and length scales: Lightning is a convection-driven local phenomenon of hourly timescale (*6*, *7*); temperature, drought, and wind conditions can move forests into highly flammable states over periods of a few days to weeks or longer (*8*); and biomass is governed by vegetation type and abundance, which regionally depend on seasonal to multi-decadal climate and can change with CO_2_ concentrations [i.e., the CO_2_ fertilization effect; (*9*)].

In turn, wildfires cause feedback effects on climate. Most natural wildfire emissions do not cause a sustained increase in atmospheric CO_2_ concentration due to vegetation regrowth (*10*), although this offset does not hold for deforestation and peatland fire emissions (*11*). However, fires do release carbonaceous aerosols and alter land surface albedo (*12*). In the atmosphere, wildfire-released aerosols affect the radiative budget both directly through scattering and absorbing of radiation and indirectly through their impacts on clouds (*13*). In addition, they alter surface albedo through deposition (*14*). Recent model experiments have demonstrated the nonlinearity in the response of yearly-to-decadal climate to fire-released aerosols (*15*–*17*). In particular, these studies have investigated differences between results from the Community Earth System Model version 2 (CESM2) when forced with prescribed biomass burning emissions input that is smoothed versus non-smoothed during the satellite period but with equivalent integrated emissions [see (*18*)]. Nonlinear sensitivities in aerosol-cloud-radiation effects cause asymmetric heating versus cooling changes between periods of low versus high aerosol burden. In the Arctic, this asymmetry promotes enhanced sea-ice melting, as well as permafrost thawing and soil drying, thus further amplifying the regional climate sensitivity to wildfire-released aerosol burden (*15*, *17*).

At the global scale, the response of lightning to anthropogenic climate change remains highly uncertain. First, from observations, most global lightning detection networks have short temporal coverage or exhibit changes in detection efficiency over time [e.g., (*19*, *20*)]. The satellite-based Lightning Imaging Sensor (LIS) covers the period 2002–2013 (*21*), which corresponds to a global warming hiatus, and no other >10-year continuous global lightning product exists. This complicates the task of associating current yearly to decadal global temperature changes with global lightning changes. Using the 2002–2013 LIS data, Williams *et al.* (*22*) derived an estimate of global lightning sensitivity to temperature of +4% K^−1^. However, this estimate is based on monthly sampling and used a yearly running mean filter, thus likely overestimating the true sensitivity over this period (*22*). Second, lightning displays a nonlinear sensitivity to aerosols, typically increasing at low aerosol loads but decreasing at high loads (*23*). This sensitivity remains challenging to quantify accurately due to the interplay of several complex processes, including microphysical processes at the scale of cloud droplets, and changes in the atmosphere vertical temperature profile through cloud-related latent heat fluxes and through radiative absorption and scattering (*23*). Last, different lightning models predict contrasting estimates of lightning sensitivity to climate warming. Estimated global-scale sensitivities typically range between −3.5 and +12% K^−1^, i.e., even the sign of the trend is uncertain (*24*, *25*).

This three-way interdependency between climate, lightning, and wildfires motivates fully coupled model experiments to better quantify this interdependency and predict its sensitivity to anthropogenic climate change. Previous studies combining a climate model with an empirical fire scheme found that, under a doubling of CO_2_ concentration and +3.5-K global temperature change, lightning and LIW burned area increase strongly, particularly in tropical forests due to more drought occurrences there (*4*, *26*). More recently, Krause *et al.* (*27*) performed Earth System model predictions combining a cloud-top-height lightning parameterization (*28*) and a wildfire model (*29*) under different climate change scenarios, although their model configuration did not account for vegetation changes and climatic impacts from wildfire-released aerosols. In their strongest warming scenario (+2.8 K, Representative Concentration Pathway 8.5), they found a +49% increase in global burned area, of which ∼5% could be attributed to large global increases in lightning frequency (*27*).

Studies combining state-of-the-art climate, lightning, and fire models in a fully coupled manner are rare. Pérez-Invernón *et al.* (*30*) have demonstrated that changes in long-continuing-current lightning flashes, i.e., flashes with a continuous current flow lasting >9 ms and hypothesized to be more likely to produce LIWs, do not necessarily follow changes in total lightning, which complicates predictions of LIW risks. But they did not use any vegetation and fire model to evaluate impacts on burned area, fire emissions, and feedback effects on regional-to-global climate. The most recent Fire Model Intercomparison Project (*31*) found a low sensitivity from six fire models to lightning changes, but their predictions used uncoupled climate, lightning, and fire-released aerosols, thus decoupling the ignition, fire weather, and biomass factors. It is also notably challenging to quantify climate-lightning-wildfire interactions only from observations due to the highly uncertain attribution of lightning- versus human-ignited wildfires (*1*), a high degree of overlap in weather conditions for wildfire igniting and non-igniting lightning strokes (*32*), and the conversion from measured wildfire-released aerosol properties to radiative effects (*33*).

To this day, many components of the three-way interactions between climate, lightning, and wildfires remain poorly quantified, as well as their sensitivity to global climate change forcing. This includes changes in lightning frequency, the relative sensitivities of wildfires to lightning and weather changes, and the global climate feedback response to realistic variability in aerosols released from wildfires. This study presents a thorough model-based investigation of such interactions. We use CESM2 (*34*), which we modified such that wildfire-released aerosols are passed directly to the atmospheric model component. Furthermore, a recent improved lightning model is coupled online with the atmospheric model (*35*) and passes lightning rates as a wildfire ignition source to the land model. Our model simulations include two idealized climatic scenarios: one under preindustrial conditions and one under a 1% yearly increase in atmospheric CO_2_ concentrations. Our goal is to investigate how such an external radiative forcing causes changes in the global and regional lightning rates. In turn, we study the specific sensitivities of wildfires from changes in lightning ignition sources, fire weather, and biomass. While our analysis is necessarily limited by its purely model-based nature, it allows identifying and quantifying key interactions using state-of-the-art climate, lightning, and fire models.

## RESULTS

Our modeling framework uses full coupling of state-of-the-art models for wildfire ignition and spread (*36*, *37*), aerosol microphysical and radiative properties (*38*), and lightning flash rate density (*35*) (see Materials and Methods). We first perform a 345-year spin-up run in which preindustrial external forcings are prescribed, such as greenhouse gas concentrations, aerosol emissions other than from biomass burning, solar activity, population density, and land use. The objective of this spin-up run is to reach a quasi-equilibrium in the mean climate state (fig. S1). This allows identifying deviations from the mean state in the subsequent transient simulations as external forcings are modified (see Materials and Methods).

We perform a set of four different transient simulations of 140 years (Table 1), all branching from the final year of the spin-up run. The purpose of these simulations is to examine the sensitivity of climate-lightning-wildfire interactions to increasing atmospheric CO_2_ concentrations, as well as the specific sensitivity of wildfires to climate-driven lightning changes. The first simulation, referred to as preindustrial lightning-on, is simply the continuation of the spin-up run for 140 years, i.e., all forcings and model configurations remain unchanged. In the second simulation, we deactivate the lightning model and, instead, prescribe a lightning climatology as forcing for wildfire ignitions; this simulation is referred to as preindustrial lightning-clim. Comparing the first and second simulations, therefore, enables an investigation of the impact on wildfire activity of uncoupling weather and lightning ignition sources. The lightning-on versus lightning-clim distinction between the third and fourth simulations is similar, but both simulations are forced with CO_2_ concentrations increasing by 1% per year following standard modeling experiment protocols (*39*); they are referred to as 1% CO_2_ lightning-on and 1% CO_2_ lightning-clim. The 1% CO_2_ year^−1^ increase corresponds to a quadrupling of CO_2_ concentrations (4 × CO_2_) at the end of the 140 model transient years. We emphasize that our simulations only capture the transient response of the climate system to +1% CO_2_ year^−1^ forcing and do not represent the climate at equilibrium with a fixed 4 × CO_2_ forcing. Also, while the CO_2_ concentration changes, other anthropogenic effects such as fire suppression/ignition, land-use change, and non–fire aerosol emissions are maintained fixed at preindustrial levels. As such, our 1% CO_2_ runs isolate the impacts from global warming-driven changes in lightning, fire weather, and biomass on the wildfire response, and the subsequent radiative perturbations through aerosol feedback, without any impacts from socioeconomic changes. See Materials and Methods for all details about the simulation setup.

**Table 1.** Configuration of simulations. The four 140-year transient simulations performed in this study.

Label	CO_2_ forcing	Lightning forcing
Preindustrial Lightning-on	Preindustrial level (284.7 ppm)	Interactive lightning model
Preindustrial Lightning-clim	Preindustrial level (284.7 ppm)	Observed climatology
1% CO_2_ lightning-on	Gradual increase of 1% year^−1^	Interactive lightning model
1% CO_2_ lightning-clim	Gradual increase of 1% year^−1^	Observed climatology

### Forced global-scale changes

**Fig. 1.**
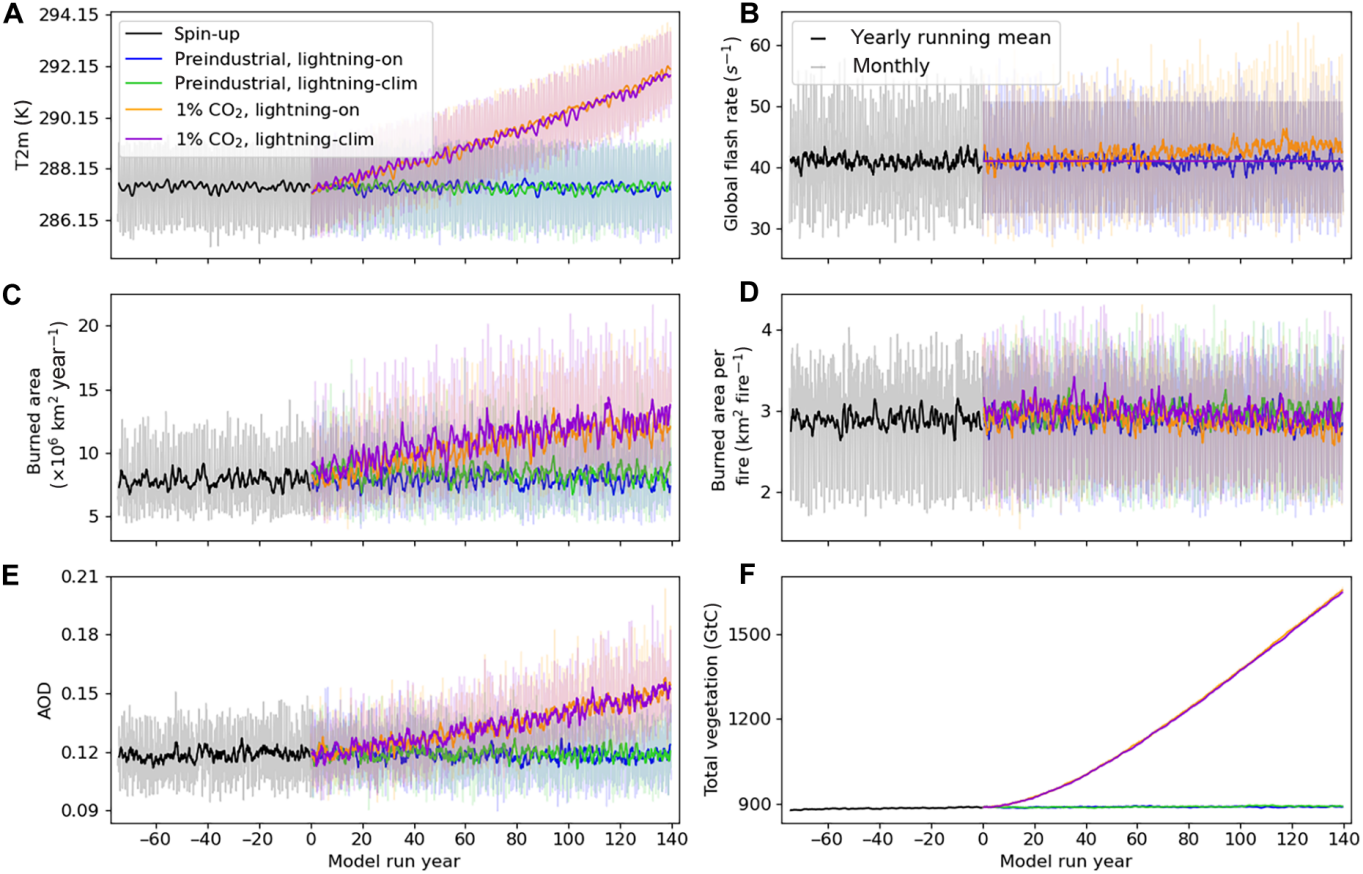
Evolution of global-scale climate indices. Yearly running mean (thick lines) and monthly values (thin lines) of (**A**) mean 2-m temperature, (**B**) total lightning flash rate, (**C**) total burned area, (**D**) mean burned area per fire, (**E**) mean aerosol optical depth (AOD), and (**F**) total vegetation carbon stocks, all at the global scale. Black curve shows the end of the spin-up run. Colored curves show the four transient simulations. Model run years are centered at 0 at the start of the transient runs. Note in (B) that the lightning-clim runs have a prescribed lightning climatology and, thus, a flat yearly running mean by design of the simulations. Note that (E) shows the AOD at 550 nm.

Figure 1 shows the evolution of key simulated global climate indicators. Relative to preindustrial, the 1% CO_2_ runs result in a global mean 2-m temperature (T2m) increase of +3.6 K, when averaged over the past 40 years of the transient simulations (Fig. 1A). There are associated global increases in total vegetation carbon stocks (+68.6%, i.e., the total amount of carbon stored in living biomass of plants), burned area (+50.2%), and mean aerosol optical depth (AOD; +23.1%) (Fig. 1, C, E, and F). Those rates of increase are very similar between the lightning-on and lightning-clim runs (orange and purple curves in Fig. 1, respectively). The four runs show a similar mean burned area per fire (Fig. 1D). This indicates no substantial mean fire size change, and, therefore, the increase in burned area is driven by a larger number of fires, which is the other component of the total burned area. Comparing the lightning-on preindustrial and 1% CO_2_ runs, we find a small global lightning increase of 2.54 flashes s^−1^ (+6.2%, Fig. 1B), but with high statistical significance (*P* < 10^−6^). This amounts to a linear sensitivity of global lightning to temperature of +1.6 ± 0.1% K^−1^, where ± denotes the linear coefficient standard error (see fig. S2).

**Fig. 2.**
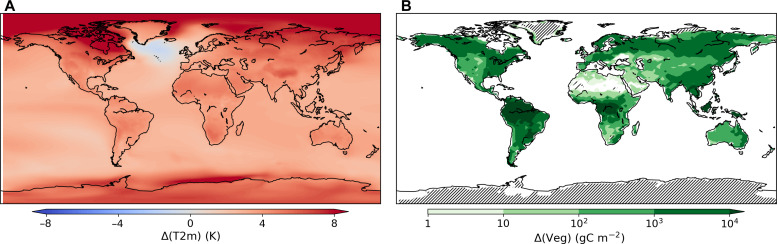
CO_2_-forced temperature and vegetation changes. Annual mean difference between the 1% CO_2_ and preindustrial runs, averaged over the past 40 years of simulation, shown for (**A**) 2-m temperature and (**B**) total vegetation carbon stocks. Note that both the lightning-on and lightning-clim runs have been pooled to increase the sample size. Hatching denotes annual mean difference not significant, evaluated with a two-tailed *t* test by controlling for an FDR α_FDR_ = 0.05 (see Materials and Methods). Note the logarithmic color scale in (B).

Figure 2 shows global maps of differences in T2m and total vegetation averaged over the past 40 years of simulation between the 1% CO_2_ and the preindustrial scenarios. As expected, temperature increases are larger in the polar regions and, on average, larger over land (+4.8 K) than over the oceans (+3.2 K). We also note the presence of the North Atlantic warming hole displaying a negative T2m change, which is statistically significant [*P* values satisfy a false discovery rate (FDR) α_FDR_ = 0.05, see Materials and Methods]. The latter feature is common in climate models and observed in historical data (*40*). Concerning total vegetation, there is a strong response to the CO_2_ fertilization effect (Fig. 2B), which is most pronounced in the equatorial forests and, to a lesser extent, in boreal forests.

### Lightning sensitivity to global warming

**Fig. 3.**
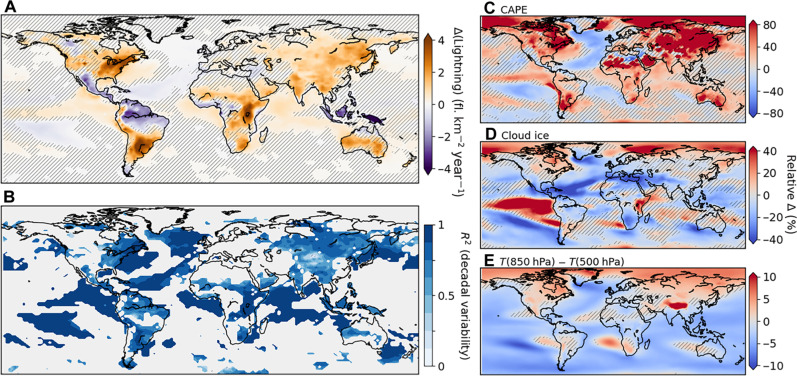
Lightning response to global warming. (**A**) Annual mean difference in lightning flash rate density between the lightning-on versions of the 1% CO_2_ and preindustrial runs, averaged over the past 40 years of simulation. (**B**) Proportion of decadal variance in lightning explained by simple three-variable linear regressions (see text for details). Areas with no significant lightning change [i.e., hatched in (A)] are not considered (grayed out). *R*^2^ denotes the coefficient of determination. The right column shows annual mean differences computed as in (A), but for three key lightning-related variables: (**C**) convective available potential energy (CAPE), (**D**) cloud ice content, and (**E**) vertical temperature gradient between the 850- and 500-hPa levels; and differences are shown relative to preindustrial run values. Note that the color bars in (C) to (E) span different ranges. Note also that the three variables shown are those used by the linear models shown in (B). Hatching denotes [(A), (C), (D), and (E)] annual mean difference not significant evaluated with a two-tailed *t* test by controlling for α_FDR_ = 0.05. In (B), the regression models are statistically significant over 99% of grid cells, as evaluated with an *F* test by controlling for α_FDR_ = 0.05.

We compare the past 40 years of simulation from the lightning-on versions of the preindustrial and 1% CO_2_ runs to analyze changes in lightning caused by the global warming signal. Across regions, we find a very heterogeneous response of lightning to the forced global warming (Fig. 3A). Over the continents, some regions of lightning increase stand out: eastern Africa, East and Southeast Asia, parts of North America, and southern South America. In contrast, equatorial regions, where lightning rates are highest (fig. S3), are mostly characterized by lightning decreases. This is clearly visible in the Amazon and the Maritime Continent, while equatorial West Africa shows mostly nonsignificant changes or only slight decreases. Last, the decrease in the Amazon also extends into Central America. There are also contrasting lightning responses to global warming over the oceans (Fig. 3A), where lightning rates are typically an order magnitude lower than over continents (fig. S3). In the equatorial oceans and most of the Indian ocean, we find small but significant increases, while the southern central Pacific shows decreases. The North Atlantic displays a horseshoe pattern: Lightning increases in the West but decreases elsewhere.

To better understand this heterogeneous lightning response to the high CO_2_ conditions, Fig. 3 (C to E) shows relative changes in three lightning-related climate indices: convective available potential energy (CAPE), cloud ice content, and the atmospheric vertical temperature gradient. CAPE measures the capacity for sustained upward air movement and is well-known to strongly influence lightning activity (*6*). Cloud ice is key because collisions between ice particles lead to charge separation and cloud electrification [the noninductive charging mechanism; (*41*)]. Last, the vertical temperature gradient characterizes the overall stability of the atmosphere. Figure 3C shows that increases in continental lightning over the midlatitudes are mostly driven by increases in CAPE. Unchanged to slightly negative changes in CAPE in Europe, around the Mediterranean, and in Central America also explain the zero to negative lightning changes in these regions. Over the oceans as well, we find a very close correspondence between the sign of change for lightning and CAPE (compare Fig. 3A and Fig. 3C), and the increase of lightning with CAPE in the convection-prone equatorial regions agrees with previous studies (*42*). However, CAPE does not seem to explain the lightning change patterns of the continental equatorial regions, motivating to expand our analysis to total cloud ice content changes (Fig. 3D). East Africa is characterized by very large increases in cloud ice (up to 40%), explaining the strong lightning increases there. In contrast, cloud ice decreases in equatorial West Africa and even more so in the Amazon, Central America, and the Maritime Continent, which leads to significant lightning decreases. Furthermore, at the global scale, we find a change in the vertical temperature gradient through the atmosphere. Figure 3E shows this gradient between 850 and 500 hPa. While it increases at high latitudes, it decreases over most of the tropics, which are the high lightning regions (fig. S3). This causes a more stable atmospheric temperature profile, less favorable to lightning. Notably, as illustrated by the New Guinea island, the lightning reductions over the Maritime Continent appear particularly sensitive to the local vertical temperature gradient decrease, which is slightly stronger there compared to the two other tropical lightning chimneys, i.e., the Amazon and central Africa (Fig. 3E). These opposing and regionally varying effects from different climate variables result in the relatively small global lightning sensitivity to global mean temperature that we find (+1.6 ± 0.1% K^−1^). Furthermore, analyzing the residuals from this sensitivity in each 140-year lightning-on simulation supports the null hypothesis for a linear sensitivity (fig. S2).

Last, we quantitatively evaluate how these three climate indices contribute to the simulated lightning difference between the 1% CO_2_ and the preindustrial run. At all grid cells with a statistically significant (*P* satisfies α_FDR_ = 0.05) lightning difference, we fit a three-variable linear model using CAPE, total cloud ice, and the 850- to 500-hPa temperature gradient. Locally, lightning displays large monthly and interannual variability, which the simple linear models do not aim to reproduce. Instead, we focus here on evaluating the causes of long-term changes. For this reason, we fit the three-variable linear models to the decadal means of both the preindustrial and 1% CO_2_ runs, i.e., including the decades of significant lightning differences. Figure 3B shows how much of the decadal variability is captured by the three-variable models [coefficient of determination (*R*^2^)]. The models are statistically significant over >98% of grid cells (*F* test, *P* satisfies α_FDR_ = 0.05). The area-weighted *R*^2^ of the three-variable models is 0.82 ± 0.17. Thus, the combined changes in CAPE, total cloud ice, and 850- to 500-hPa temperature gradient explain a majority of the decadal lightning changes in our model experiments, even under the assumption of simple linear relationships without interaction effects. Note that our interpretation of the lightning sensitivity is supported by our evaluation of modeled lightning spatial patterns, variability, and seasonal climatology against the combined OTD and LIS satellite data product (fig. S3).

### Wildfire response to climate, vegetation, and lightning changes

**Fig. 4.**
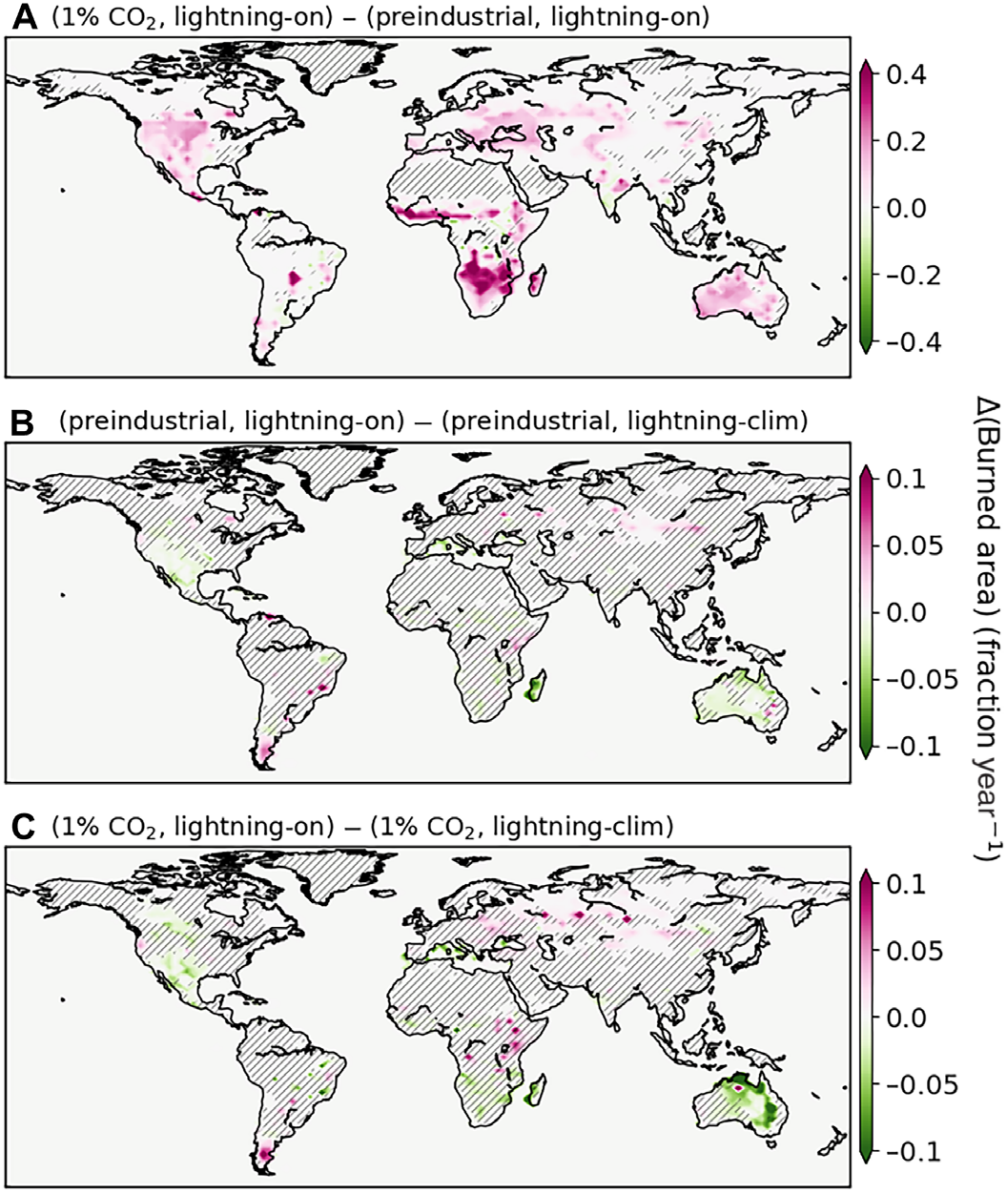
Burned area changes. Annual mean difference in fractional burned area averaged over the past 40 years of simulation (**A**) between the 1% CO_2_ and preindustrial lightning-on runs, (**B**) between the preindustrial lightning-on and lightning-clim runs, and (**C**) between the 1% CO_2_ lightning-on and lightning-clim runs. Note that the color bar in (A) spans four times the range of the color bars in (B) and (C). Hatching denotes annual mean difference not significant, evaluated with a two-tailed *t* test by controlling for α_FDR_ = 0.05.

The +50.2% (+4.1 × 10^6^ km^−2^ year^−1^) global increase in burned area in the 1% CO_2_ runs corresponds to a linear sensitivity to global mean temperature of +13.8 ± 0.3% K^−1^, where ± denotes the linear coefficient standard error (fig. S4). Here, also, residuals from a linear fit support the null hypothesis of a linear sensitivity to temperature (fig. S4). It is important to remember that this sensitivity includes both the climatic and CO_2_ fertilization effects but excludes the direct anthropogenic influences. The global distribution of differences in burned area between the 1% CO_2_ and preindustrial lightning-on runs is shown in Fig. 4A; we are focusing here also on the past 40 years of the simulations. There are widespread increases in burned area, which are particularly pronounced in Southern Africa and Australia, as well as throughout North America and eastern Europe. As shown in Fig. 2B, there is a large worldwide increase in vegetation, which provides more biomass fuel for fires. Thus, while the vegetation increase does not visually explain the spatial distribution of burned area changes (compare Fig. 2B and Fig. 4A), changes in vegetation can affect burned area by loosening the fire fuel constraint in biomass-limited fire regimes.

**Fig. 5.**
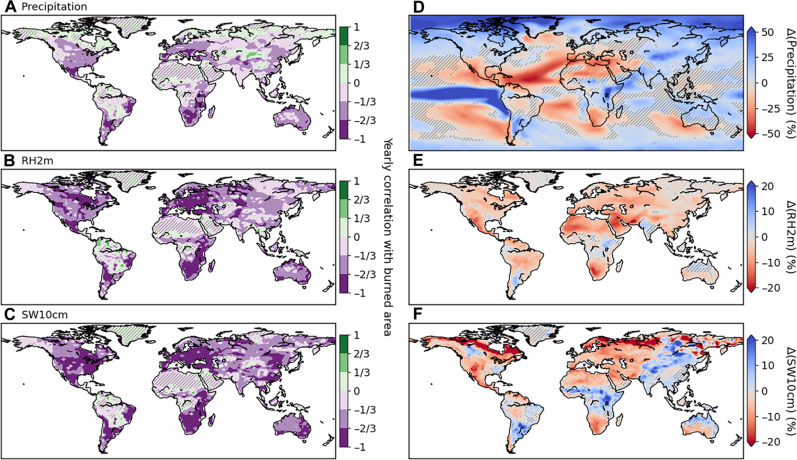
Moisture indices relation to burned area. The left column shows correlation between annual mean burned area and annual mean (**A**) precipitation, (**B**) relative humidity at 2-m height (RH2m), and (**C**) upper 10-cm soil water content (SW10cm). The right column shows annual mean differences between the 1% CO_2_ and preindustrial runs, averaged over the past 40 years of simulation for (**D**) precipitation, (**E**) RH2m, and (**F**) SW10cm; and differences are shown relative to preindustrial run values. Note that the color bar in (D) spans 2.5 times the range of the color bars in (E) and (F). For correlations [(A) to (C)], all runs have been pooled, and for differences [(D) to (F)], lightning-on and lightning-clim runs have been pooled to increase the sample size. Hatching denotes [(A) to (C)] correlation or [(D) to (F)] annual mean difference not significant, evaluated with a two-tailed *t* test by controlling for α_FDR_ = 0.05.

Focusing on variables controlling fire weather, we show changes in annual precipitation, 2-m relative humidity (RH2m), and upper 10-cm soil water content (SW10cm) (Fig. 5). We pool together all four transient simulations to compute yearly correlation between the fire weather variables and burned area, shown in the left column of Fig. 5. There is a widespread negative correlation between these moisture indices and fires. Only precipitation shows significantly positive correlation in high-latitude areas (Fig. 5A), as enhanced precipitation there is generally accompanied by warmer temperatures and increased biomass availability. The right column of Fig. 5 shows the differences in the fire weather variables between the 1% CO_2_ and preindustrial runs, averaged over the past 40 years of simulation. Precipitation changes (Fig. 5D) show some degree of correspondence with areas of fire changes (Fig. 4A). For example, there is a clear decrease in Southern Africa and Mexico, two regions where the negative correlation with burned area is pronounced (Fig. 5A). On the other hand, precipitation changes do not explain burned area changes in many regions, such as North America and Australia. In these regions, the combination of negative correlation and significant decreases in both RH2m and SW10cm drives the burned area increases. It can be noted that, apart from permafrost regions, changes in RH2m and SW10cm are generally similar, but not in eastern Asia, where SW10cm shows nonsignificant or positive changes. This likely explains the smaller fire increases in eastern compared to western Eurasia. Last, we note that precipitation, RH2m, and SW10cm all increase in East Africa, which, nevertheless, experiences a high increase in burned area. This increase cannot be attributed to the lightning changes there, as differences between the lightning-on and lightning-clim runs are not significant (Fig. 4, B and C). This indicates that fires in East Africa are biomass limited in the preindustrial scenario, and the vegetation gains there under the 1% CO_2_ forcing cause the simulated fire increase. To a lesser extent, the same analysis holds for northern Australia, with nonsignificant or positive changes in moisture indices (Fig. 5) yet an increase in burned area (Fig. 4A).

To evaluate the impact of using an online atmosphere-driven lightning model versus a lightning climatology on burned area, we analyze the differences between the lightning-on and lightning-clim runs, both for the preindustrial and 1% CO_2_ scenarios. At the global scale, total burned area and global mean AOD from those runs are close (Fig. 1, C and E). However, under both climate forcing scenarios, we do find a significantly smaller annual mean total burned area in the lightning-on run compared to the lightning-clim, by −4.5% in the preindustrial scenario and −5.0% in the 1% CO_2_ scenario (both with *P* < 0.01, two-sided *t* test on annual total values over the past 40 years of simulation). Analyzing also global mean burned area per fire, Fig. 1D indicates small differences between the runs. Pooling the preindustrial and 1% CO_2_ lightning-on runs versus lightning-clim runs, we find again a small but significant difference of −6.1% smaller fires when using the online-coupled lightning model (*P* < 10^−4^, same test). This difference is approximately twice as large than when comparing 1% CO_2_ versus preindustrial runs (−3.3%, *P* < 0.01, same test), which is computed by pooling the lightning-on and lightning-clim runs. As such, using an online lightning model affects mean individual fire area almost twice as much as the high CO_2_ forcing in our simulations.

Spatial differences in burned area averaged over the past 40 years of simulation between the lightning-on and lightning-clim runs are shown in Fig. 4 (B and C), thus showing the impact from the online lightning model on burned area. In general, we find only minor differences between the lightning-on and lightning-clim runs, even at the local level. However, a few regions display statistically significant differences. Notably, the preindustrial and 1% CO_2_ scenarios agree on most of those regions. This indicates not only that the differences are meaningful beyond coincidence but also that fires in these specific regions are sensitive to the synchronization between interannual variability of lightning and fire weather under different background climate states. The regions concerned include parts of northern central Asia, southern North America, the southern tip of South America, and central Australia. In the 1% CO_2_ run, Siberia shows a significant fire-increasing impact from the use of online-coupled lightning and over an area larger than in the preindustrial (Fig. 4C). Even if the magnitude is small, we identify a correspondence with lightning increases there (Fig. 3A). We also note that the lightning-clim runs do not assume a total decoupling of lightning and fire weather because they use a prescribed 2-hourly lightning climatology, and not a constant lightning forcing (see Materials and Methods). Because a large part of the variability in fire weather variables originates from seasonality as well, it is only the climatological anomalies between lightning and fire weather that are decoupled.

### Universality of the wildfire response at the regional scale

**Fig. 6.**
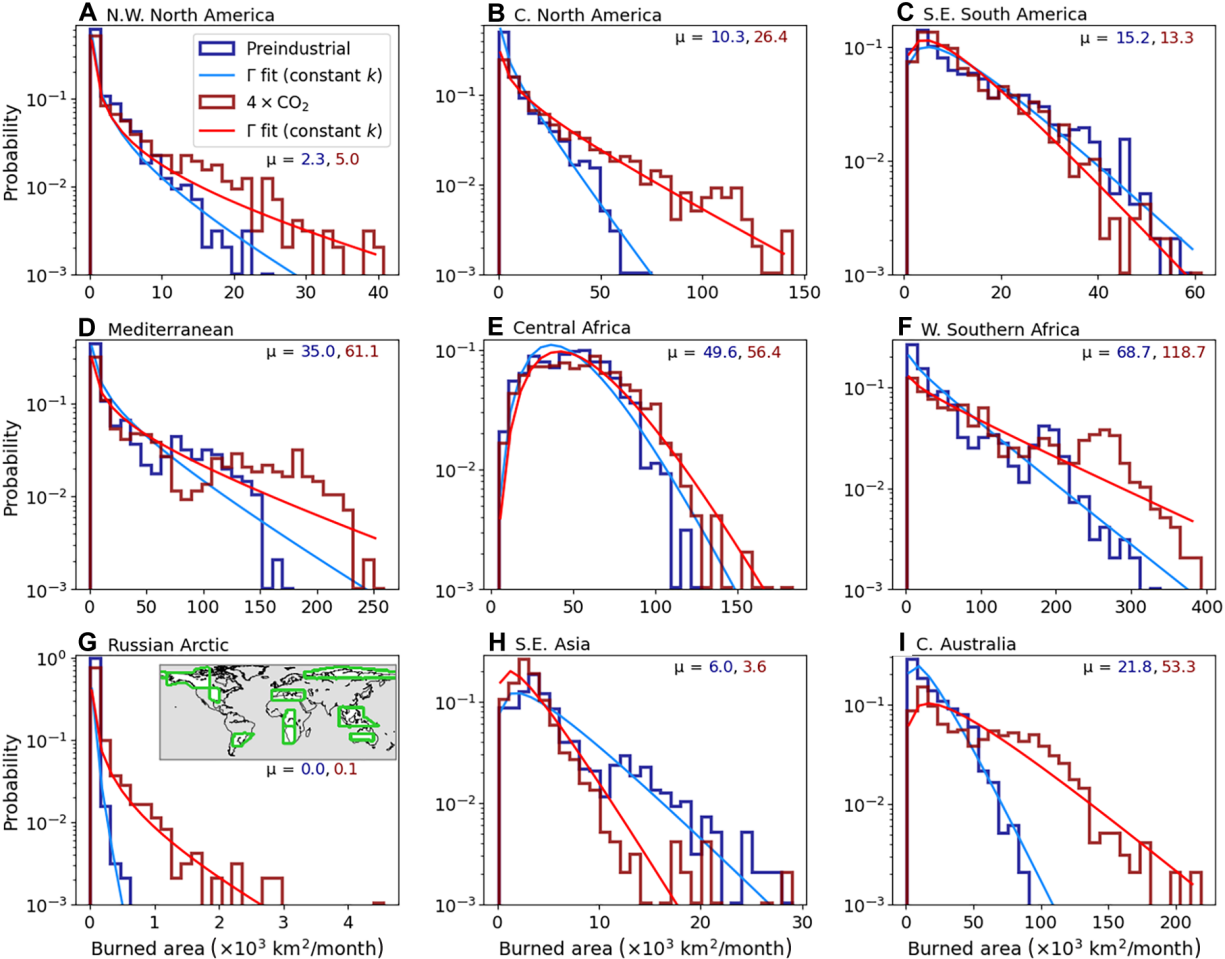
Regional-scale burned area distributions. Histograms of regional total monthly burned area over the past 40 years of simulation of the (dark blue) preindustrial runs and (dark red) 1% CO_2_ runs. Probabilities on the *y* axes are shown with a logarithmic scale. Subpanels (**A**) to (**I**) show different regions, with name given on top of each subpanel, and delineations shown in the inset map in (G). For each region and climate forcing, the sample mean (μ; units × 10^3^ km^2^ per month) is given following color coding. The thin light blue curves show the fit of analytical $\Gamma \left(k,\theta_{\text{pi}}\right)$ distributions to the preindustrial histograms. The thin light red curves show the fit of $\Gamma \left(k,\theta_{{\text{CO}}_{2}}\right)$ distributions to the 1% CO_2_ histograms but keeping the shape parameter *k* identical to the one of the corresponding preindustrial curves. Note that the lightning-on and lightning-clim runs have been pooled to increase the sample size. Nine of the 44 Intergovernmental Panel on Climate Change (IPCC) regions are shown, but similar figures for all regions are shown in figs. S6 to S10. N.W. North America, Northwestern North America; C. North America, central North America; S.E. South America, southeast South America; W. Southern Africa, West Southern Africa; S.E. Asia, Southeast Asia; C. Australia, Central Australia.

**Fig. 7.**
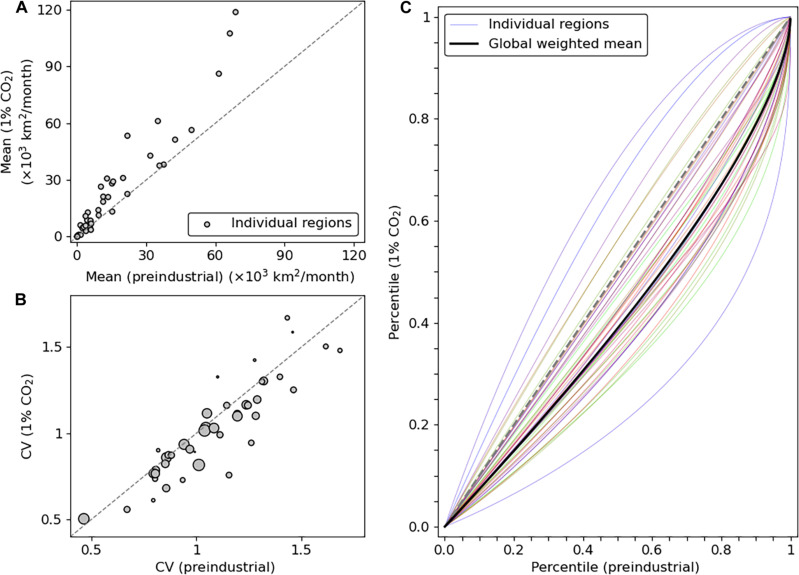
CO_2_-forced changes in statistics of monthly burned area. Changes from the preindustrial (*x* axis) to 1% CO_2_ (*y* axis) runs in (**A**) mean and (**B**) coefficient of variation (CV) of region-specific monthly total burned area. In (A) and (B), the gray scale is according to the absolute mean latitude of each region. In (B), the area of each circle is proportional to the square root of the mean total burned area. There are 44 land-based regions, shown in fig. S5. (**C**) Percent-percent plot for monthly total burned area by region. Each (*x*, *y*) pair shows the mapping from a given preindustrial percentile to the 1% CO_2_ percentile for an event of same magnitude. Percentiles are calculated analytically from the fitted Γ distributions, shown in Fig. 6 and figs. S6 to S10. The global mean (thick black curve) is weighted according to the regional mean total burned areas. In all panels, distributions are computed only over the past 40 years of simulation but pooling the lightning-on and lightning-clim runs to increase the sample size.

The burned area difference map (Fig. 4A) shows the changes in annual mean at each location caused by the 1% CO_2_ year^−1^ forcing, which exhibits spatial homogeneity at the regional level. To pursue this analysis further, we analyze the distribution shift between the preindustrial and 1% CO_2_ fire regimes. We compute monthly total burned area over the past 40 years of simulation of the preindustrial and 1% CO_2_ runs, pooling both the lightning-on and lightning-clim runs. The burned area is separated between the 44 land-based reference regions of the Intergovernmental Panel on Climate Change [IPCC; (*43*); see regions in fig. S5]. Figure 6 shows the region-specific distributions of the monthly total burned area for nine selected regions and how these distributions change under the 1% CO_2_ forcing. There is a pronounced shift of the distributions toward increased burned area for most regions (Fig. 6; see figs. S6 to S10 for all regions). Consequently, we find an increase in the mean for 38 of the 44 regions (Fig. 7A; see table S1 for all values). The absolute increase is largest in the high-burning African regions, up to +49.9 × 10^3^ km^2^ per month in West Southern Africa (Fig. 6F). In particular, the two southern-most African regions account for 20.1% of the global burned area and for 27.0% of its increase under the 1% CO_2_ forcing. Other regions of strong relative and absolute burned area increase are western and central North America (e.g., Fig. 6B), the Mediterranean (Fig. 6D), and Australia, particularly central Australia (Fig. 6I). In contrast, tropical regions show a minor increase (e.g., Fig. 6E) or even a slight decrease in fire activity (Fig. 6, C and H), particularly in South America with two regions of declining burned area. Fire in the Arctic is often hypothesized to be strongly sensitive to climate warming (*44*). Defining the Arctic as all areas of latitude >60°N, we find a 530% increase in burned area under the 1% CO_2_ forcing (table S1), as well as pronounced changes in regional distributions (e.g., Fig. 6, A and G). While the relative increase is large, it corresponds to an absolute increase of only 0.75 × 10^3^ km^2^ per month, thus contributing to 0.2% of the global increase. This modest absolute increase is clearly visible in Fig. 7A, where points at the lower end do not exhibit a major shift toward intermediate values, which are representative of midlatitude fire activity.

Across IPCC reference regions, we find an increase not only in mean but also in variance (table S1). As such, we also analyze changes in the coefficient of variation (CV), which is the ratio of the SD to the mean (Fig. 7B). We find that, across regions, the CV generally remains close to constant from the preindustrial to the 1% CO_2_ distributions, with a burned area–weighted mean absolute change of 0.07 (Fig. 7B). The 10-to-90% range of the CV changes is (−0.21, +0.11), confirming minor CV changes across regions despite differences in vegetation types and seasonality, and the strong CO_2_ forcing applied. This proportionality property between SD and mean is characteristic of a subset of gamma distributions. A gamma distribution with shape parameter *k* and scale parameter θ is defined on the positive real domain and has its mean (μ), SD (σ), and probability density function [*p*(*x*)] defined as

$$\Gamma \left(k,\theta \right):\left\{\begin{array}{c} \mu =k\times \theta \\ \sigma =\sqrt{k}\times \theta \\ p\left(x\right)=\frac{1}{\Gamma \left(k\right)\theta^{k}}x^{k-1}\text{exp}\left(-\frac{x}{\theta }\right) \end{array}\right.$$ (1)

where boldface **Γ** denotes the gamma function. As such, any Γ with fixed *k* satisfies a strict proportionality between μ and σ. Here, we fit a Γ distribution only to the preindustrial regional-scale monthly burned area distributions, shown by the light blue curves in Fig. 6. Then, we keep the calibrated *k* value fixed and only change θ to capture the change in distributions from the preindustrial to the 1% CO_2_ case; the resulting fits are shown with the light-red curves in Fig. 6. For the large majority of regions, we find an excellent fit of the Γ distributions and for both climatic background states (Fig. 6; see figs. S6 to S10 for all regions). We note that the gamma fits and proportionality between μ and σ remain valid when applied to fire emissions instead of burned area (not shown).

These analytical fits allow investigating changes in monthly burned area probabilities, including extreme events, without being limited by sampling biases. In Fig. 7C, we show a probability-probability plot in which, for each region, the value corresponding to a given percentile in the preindustrial distribution (*x* axis) is mapped to its percentile in the 1% CO_2_ distribution (*y* axis). The gray 1:1 dashed line illustrates a hypothetical unchanged distribution. Most curves go well below this 1:1 line, indicating that the probability of exceeding a given magnitude of burned area increases for all magnitudes in the 1% CO_2_ distributions. The black curve shows the global mean percentile changes, weighted by the burned area magnitude of each region. Strong changes occur for the preindustrial median events (i.e., 0.5 percentile), which are mapped to percentiles of 0.42 ± 0.10 (mean ±1σ across regions; see table S1). Extreme events in the preindustrial become significantly more common in the 1% CO_2_ scenario: The regional 0.95 and 0.99 percentiles are mapped to 0.87 ± 0.07 and 0.95 ± 0.04, respectively. Southern Africa, which includes the regions of largest monthly mean burned area, is particularly affected, with a decrease of the 0.50, 0.95, and 0.99 percentile events to percentiles smaller or equal to 0.36, 0.85, and 0.94, respectively (table S1). This influences the burned area–weighted global mean, which displays, for example, a change of the 0.99 percentile event to the 0.95 percentile (Fig. 7C and table S1). In other words, the 1% most extreme fire month events in the preindustrial become five times more likely at the end of the 1% CO_2_ scenario.

We explore the physical reason behind the universality of the gamma-distributed burned area at the regional scale. We base this analysis on well-established statistical mechanics approaches to climate theory (*45*). Our distribution fits keep *k* fixed regardless of climatic state, while changing θ to account for the CO_2_ forcing. Gamma distributions have been used to model a wide range of phenomena, such as precipitation (*46*) and fluctuations in population dynamics (*47*). One of the underlying reasons for such a large applicability is that the gamma distribution is a steady-state solution to a growth-and-decline stochastic differential equation. Let *x* be the fire activity at a given month, thus evolving with time *t*. Assume that x* is the equilibrium fire activity level, that τ is the characteristic timescale for return to x*, and that ξ is the strength of fire activity fluctuations. One can then write a simple stochastic linear response model (*47*), which serves as a highly simplified representation of monthly burned area

$$\frac{\mathrm{dx}}{\mathrm{dt}}=\frac{x^{*}-x}{\tau }+\sqrt{2\xi x}\eta \left(t\right)$$ (2)

where η(*t*) represents Gaussian uncorrelated noise. The Fokker-Planck equation for the associated probability density function, *P*(*x*,*t*), is given by

$$\frac{\partial p\left(x,t\right)}{\partial t}=-\frac{\partial }{\partial x}\left[\left(\frac{x^{*}-x}{\tau }\right)p\left(x,t\right)\right]+\xi \frac{\partial^{2}}{\partial x^{2}}\left[xp\left(x,t\right)\right]$$ (3)

The steady-state solution, $p_{\text{ss}}\left(x\right)$, to this stochastic linear response model is obtained by setting the right-hand-side equal to zero, rearranging terms, and integrating (see Supplementary Text). The solution probability density function is

$$p_{\text{ss}}\left(x\right)∼\Gamma \left(\frac{x^{*}}{\xi \tau },\xi \tau \right)$$ (4)

That is, a gamma distribution with shape parameter $k=\frac{x^{*}}{\xi \tau }$ and scale parameter θ = ξτ. Our results show that θ generally increases in the 1% CO_2_ runs, because the mean burned area increases in the large majority of regions while fixing *k*. This could be caused by an increase in amplitude of burned area fluctuations (ξ) or time to return to equilibrium (τ). The fact that the globally averaged burned area per fire shows a weakly negative change (Fig. 1D) indicates that the former effect is driven by more anomalously high numbers of fire events per month. The increase in τ suggests that ecosystems take a longer time to return to normal fire activity, for example, due to a lengthening of the fire season. The change in monthly climatology of burned area confirms that, for most regions, both fire season duration and monthly fire intensity increase (figs. S11 and S12), suggesting changes in both τ and ξ. However, because *k* remains approximately constant regardless of climatic state, there is a compensation for the increase in ξτ through an increase in x*: the equilibrium state fire activity. This compensation results in a constant equilibrium-to-noise ratio, $\frac{x^{*}}{\xi \tau }$.

### Climate feedback: Fire aerosol impacts on radiation

The 1% CO_2_ forcing causes large changes in fire emissions (+97.7%, averaged over the past 40 years of simulation). The coupling between fire emissions and the aerosol model enables a quantitative assessment of the resulting effect on the radiative budget. We focus on the fire aerosol impacts on net shortwave radiative fluxes at the top-of-atmosphere (TOA), because this is the part of the spectrum in which aerosols scatter and absorb more effectively (*48*). Following well-established procedures (*49*), we derive the radiative forcing from the direct fire aerosol effect under all-sky conditions, $F_{\text{all},f}^{\left(d\right)}$, which quantifies the total contribution to the radiative budget from scattering and absorption. In addition, we compute the same effect but under clear-sky conditions only, $F_{\text{clear},f}^{\left(d\right)}$, which allows us to isolate a residual nonlinear term, $F_{\text{res},f}^{\left(d\right)}=F_{\text{all},f}^{\left(d\right)}-F_{\text{clear},f}^{\left(d\right)}$. The latter component represents the additional direct fire aerosol effect when conditions deviate from clear-sky to all-sky conditions, and it results from the complex combined influence of fire aerosols and clouds on the radiative forcing (see Materials and Methods). However, we emphasize that all $F_{\cdot ,f}^{\left(d\right)}$ terms account for the direct aerosol effect from fire aerosols only (f subscript), as our analysis procedure separates this component from those associated to other aerosols (see Materials and Methods).

**Fig. 8.**
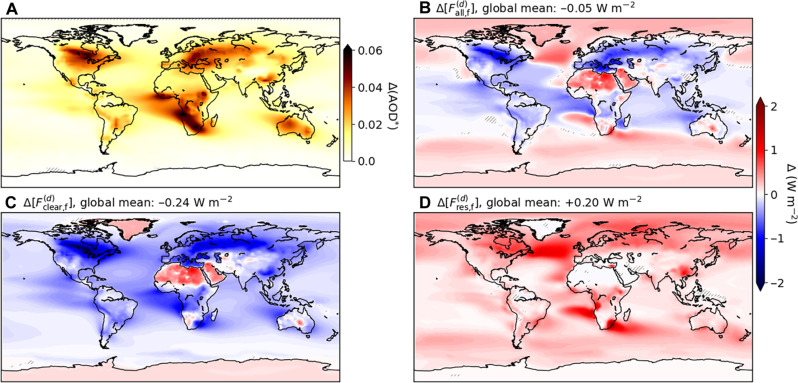
Fire-induced changes in direct aerosol effect. Annual mean difference averaged over the past 40 years of simulation between the 1% CO_2_ and preindustrial runs for (**A**) fire aerosol component of AOD changes [Δ(AOD*), dimensionless], the radiative forcing from the direct fire aerosol effect (**B**) under all-sky conditions $\left\{\Delta \left[F_{\text{all},f}^{\left(d\right)}\right]\right\}$, and (**C**) under clear-sky conditions, $\left\{\Delta \left[F_{\text{clear},f}^{\left(d\right)}\right]\right\}$, and (**D**) the difference between (B) and (C), $\left\{\Delta \left[F_{\text{res},f}^{\left(d\right)}\right]\right\}$. Global area-weighted mean values for (B) to (D) are given on top of their subpanel. Note that the lightning-on and lightning-clim runs have been pooled to increase the sample size. Note also that AOD* in (A) is taken as the AOD at 550 nm and excludes contributions from variations in non–fire aerosol emissions (see Materials and Methods). Hatching denotes annual mean difference not significant, evaluated with a two-tailed *t* test by controlling for an FDR $\alpha_{\text{FDR}}=0.05$.

Figure 8A shows the differences between the preindustrial and 1% CO_2_ runs in AOD*, which we define as the component of AOD representing wildfire-released aerosol variability (see Materials and Methods), and differences are averaged over the past 40 years of simulation. The largest changes in AOD* coincide with those in burned area (compare Figs. 4A and 8A). For example, there is a large increase in AOD* over Southern Africa, caused by the large absolute change in fire emissions there. This feature then propagates with the dominating easterlies over the Atlantic Ocean. More generally, atmospheric transport causes a global-scale increase in AOD*, even over non–fire-prone areas, which is small in most areas but highly significant (Fig. 8A). Concerning total AOD, dust emissions increase over northern Africa but decrease in central Asian deserts due to enhanced vegetation, and sea salt emissions increase over most of the ocean regions due to oceanic warming (fig. S13).

Focusing on the radiative fluxes, we compute the difference between the 1% CO_2_ and preindustrial runs in the same manner for the three components $F_{\text{all},f}^{\left(d\right)}$, $F_{\text{clear},f}^{\left(d\right)}$, and $F_{\text{res},f}^{\left(d\right)}$: $\Delta \left[F_{\text{all},f}^{\left(d\right)}\right]$, $\Delta \left[F_{\text{clear},f}^{\left(d\right)}\right]$, and $\Delta \left[F_{\text{res},f}^{\left(d\right)}\right]$, respectively. Under clear-sky conditions (Fig. 8C), the AOD* increase through increased fire emissions mostly causes more scattering, and thus decreased $F_{\text{clear},f}^{\left(d\right)}$, in agreement with previous work (*12*, *13*). This effect is reduced or even reversed in the polar regions, where the total radiative forcing from the direct fire aerosol effect is dominated by black carbon (*50*), which has strong absorption characteristics. This is more pronounced over grounded ice sheets than sea ice, mostly because concurrent sea-ice thickness and extent reductions change the background albedo, and thus the relative importance of aerosol scattering in the radiative budget (*51*). Values of $\Delta \left[F_{\text{clear},f}^{\left(d\right)}\right]$ are also positive over desert regions (Fig. 8C), due to relatively high surface albedo and to the high background in dust aerosols that reduces the relative contribution of fire aerosols to scattering. The patterns of $\Delta \left[F_{\text{all},f}^{\left(d\right)}\right]$ are more heterogeneous (Fig. 8B), demonstrating the complexity of impacts from concurrent changes in clouds and fire aerosols in the 1% CO_2_ runs. For example, a region with increased cloud coverage would, all other things being equal, undergo an increase in $F_{\text{all},f}^{\left(d\right)}$ because the enhanced cloud cover would reflect part of the flux previously scattered by aerosols. The anomalously high positive values found on the South-West African coast and in the North Atlantic (Fig. 8B) follow this logic, as these are regions of enhanced cloud coverage (fig. S14). There is a general contrast in $\Delta \left[F_{\text{all},f}^{\left(d\right)}\right]$ along latitudes: positive at high latitudes but generally negative at mid- and low latitudes. This implies that increased fire activity, driven by global warming, causes a direct aerosol radiative forcing with a warming impact at high latitudes and cooling impact at low latitudes. As a result, this fire-related direct aerosol radiative forcing contributes to polar amplification, but the total aerosol effect would also depend on the indirect and surface albedo effects [e.g., (*52*, *53*)]. Last, the map of $\Delta \left[F_{\text{res},f}^{\left(d\right)}\right]$ is, by definition, the difference between $\Delta \left[F_{\text{all},f}^{\left(d\right)}\right]$ and $\Delta \left[F_{\text{clear},f}^{\left(d\right)}\right]$ (Fig. 8D). Still, it demonstrates that the 1% CO_2_ year^−1^ forcing causes a ubiquitous increase in $F_{\text{res},f}^{\left(d\right)}$. This aspect should not be overlooked, as it implies that the negative contribution of the direct fire aerosol effect is more reduced under all-sky compared to clear-sky conditions in a high-CO_2_ climate than it is in a preindustrial climate. Maps of $F_{\text{all},f}^{\left(d\right)}$, $F_{\text{clear},f}^{\left(d\right)}$, and $F_{\text{res},f}^{\left(d\right)}$ in the preindustrial and 1% CO_2_ runs are shown in fig. S15.

Global area-weighted mean values of each flux change are also given in Fig. 8. We find an overall negative change in mean radiative forcing from the all-sky direct fire aerosol effect of −0.05 W m^−2^. This corresponds to an absolute increase, i.e., more negative, in the direct fire aerosol effect of 19.8% relative to the preindustrial mean. Concerning the polar amplification identified above, the mean $\Delta \left[F_{\text{all},f}^{\left(d\right)}\right]$ at latitudes equator- versus poleward of 60° are −0.072 and +0.106 W m^−2^, respectively. It is also insightful to compare the global mean contribution from fire aerosol radiative forcing to the net total TOA shortwave flux (fig. S16). With respect to this total metric, the global mean of −0.05 W m^−2^ forcing change corresponds to a −0.02% change relative to the preindustrial mean. Regionally, this contribution varies from −0.60% in North-East North America and central Eurasia to +0.35% in the North Atlantic and +0.50% in the Arctic (fig. S16). Under clear-sky conditions (Fig. 8C), the global mean change in $\Delta \left[F_{\text{clear},f}^{\left(d\right)}\right]$ is 5.0 times more negative. This means that, in the 1% CO_2_ runs, the differences between all-sky and clear-sky conditions amplify compared to the preindustrial conditions. In other words, there is an increasing compensation through cloud-modulated processes of the radiative forcing from the direct fire aerosol effect, which strongly reduces the global average impact of the increase in fire-released aerosols. Such processes include changes in the cloud distribution itself, as well as absorption from aerosols overlaying clouds (*12*, *51*).

## DISCUSSION

From our simulations, we derive a significant positive sensitivity of total lightning to global mean temperature of +1.6 ± 0.1% K^−1^. This estimate falls between those from previous model experiments. In particular, previous estimates from models based on purely convective climate variables such as CAPE and cloud-top-height are much larger [+5 to 12% K^−1^; (*4*, *25*)]. In contrast, those based only on cloud ice flux are negative [−3.5% K^−1^; (*24*)]. Here, using a more complex lightning model based on 12 climate variables (*35*), our global estimate falls in-between those previous contrasting approaches. This has a physical basis that highlights the importance of a more comprehensive lightning model: On decadal timescales, continental lightning increases over the midlatitudes driven by CAPE but decreases in most equatorial regions, caused by a decrease in cloud ice availability and a more stable vertical temperature gradient. Equatorial East Africa is an exception with the largest lightning increase, which is driven by cloud ice gains. It is, nevertheless, important to note that cloud and convection variables are sensitive to the choice of convection scheme (*54*), and both the lightning change amplitude and patterns that we identify should be explored using other climate models and convection configurations. We note that, although their observational estimate is influenced by monthly temporal sampling and serial correlation from a running mean, our global sensitivity estimate (+1.6 ± 0.1% K^−1^) is within the 95% confidence interval reported by Williams *et al.* (*22*) [+(0.6; 7.4)% K^−1^], particularly considering that their interval is “biased high” (*22*). Last, the sensitivity and uncertainty estimates that we find are directly relevant to the evaluation of climate feedback processes through greenhouse gas forcing, because the effects from lightning NO*_x_* emissions are a major uncertainty in current budgets and future predictions of tropospheric ozone and methane (*24*, *55*).

At the global scale, the impact of changing lightning on wildfire activity is small in our simulations. Although of low magnitude (−4.5 to −5.0%), the difference in annual global total burned area is significant when using an online lightning model versus a lightning climatology (*P* < 10^−2^) under both preindustrial and high-CO_2_ climate. Similarly, we find a low (−6.1%) but significantly (*P* < 10^−4^) smaller average fire size with the online lightning model. The lightning climatology leads to overestimated burned area and fire size, caused by a decoupling of interannual variability in fire weather and ignition timing and thus removing some correlation between lightning and precipitation for example. We find agreement between the preindustrial and 1% CO_2_ runs on a few regions sensitive to lightning variability. These regions are larger in the 1% CO_2_ run, indicating more extensive regulation of burned area through lightning in high-CO_2_ conditions. One prominent case is Siberia, where increased lightning drives part of the increase in wildfire activity under the 1% CO_2_ year^−1^ forcing. However, burned area in other Arctic regions such as far-East Russia, Alaska, and North-East Canada appear mostly insensitive to comparable lightning increases (Figs. 3 and 4). As such, our model results do not support empirical hypotheses of lightning-limited Arctic fire regimes, with the expectation that fire activity increases strongly with lightning over multi-annual to multi-decadal timescales (*1*, *44*). Instead, in our simulations, the Arctic remains a low-burning region, despite the prescribed 1% CO_2_ year^−1^ forcing. For example, after 100 to 140 years with this forcing level, the mean burned area in the Arctic (latitudes higher than 60°N) is still >50 times smaller than in the latitude band 50°N to 60°N, despite their land areas being equal within 1%. This low absolute increase in Arctic fire activity is mostly caused by only weak decreases in relative humidity (Fig. 5E), which remains very high, even during the fire season (fig. S17); the critical impact of relative humidity on Arctic fire activity has also been confirmed in observational studies (*56*). Although simulated Arctic wildfires likely exhibit some degree of model dependence, we note that low lightning sensitivity and small absolute wildfire changes in the Arctic are found consistently across fire models (*27*, *31*, *57*, *58*). Nevertheless, this could also point to shared model limitations; for example, the fire model used here does not simulate multiday fires (*36*), which can affect fire activity estimates in the Arctic and elsewhere. Furthermore, our lightning model does not discriminate between dry-lightning and other lightning flashes, and dry-lightning variability could have a larger impact on lightning-driven changes in wildfire activity (*59*). The importance of quantifying uncertainty in the sensitivity of Arctic fires to climate change also highlights the need for longer and more extensive fire and lightning observational records there (*3*). In particular, lightning has been observed to increase in the Arctic over the past decade (*60*), and it is critical to quantify the sensitivity of Arctic wildfires to this lightning increase relative to other features of Arctic climate change.

By ignoring changes in anthropogenic factors, our results isolate the response of wildfire activity to the CO_2_ forcing. By design of our experiments, this includes absence of changes in deforestation fires and, thus, low burned area rates in tropical closed forests (see Material and Methods and fig. S3). We derive a linear sensitivity of global total burned area to global warming of +13.8 ± 0.3% K^−1^ (fig. S4), but we emphasize that this response includes sensitivity to changes in fire weather, as well as increased biomass availability due to the CO_2_ fertilization effect (*9*). Our burned area sensitivity estimate is close to the estimated +14.5% K^−1^ from a very recent multi-model ensemble performed over the historical period (*57*). Because they use a distinct method, i.e., they subtract results of constant-climate simulations to historical climate-changing simulations to remove the anthropogenic fire modulation, the good agreement of both results is encouraging. This may indicate a method-agnostic consensus on the total burned area specific sensitivity to changing CO_2_ concentration and climate (*57*). Also, our study uses a larger CO_2_ forcing (4 × CO_2_) and, thus, global warming signal (+3.6 K over 140 years) compared to theirs [+1.1 K over 118 years; (*57*)], which indicates that the linear sensitivity to temperature change is a valid null hypothesis, as supported by the residuals from the trend that we derive (fig. S4). In addition, we note that our sensitivity of global fire emissions (+26.9 ± 0.4% K^−1^) is approximately double that of burned area (+13.8 ± 0.3% K^−1^), as a result of larger biomass burning density. Now, climate model projections generally use prescribed input data sets of fire emissions (*18*, *34*). These prescribed emissions account for future changes caused only by socioeconomic factors (*61*). However, our results demonstrate that impacts from changes in fire weather and CO_2_ fertilization are large. If future modeling studies do not calculate fire emissions online as done in this work, then future fire emission input data should incorporate estimates of such non-socioeconomic drivers as well.

In terms of radiative forcing, we find a negative feedback loop: Higher CO_2_ concentrations cause an increase in fire aerosol emissions, which decreases the global mean net shortwave TOA radiative forcing through the direct aerosol effect. In our 1% CO_2_ runs, averaging over the simulation period 100 to 140 years, this negative feedback amounts to −0.05 W m^−2^ globally. While this impact is generally negative in low and midlatitudes, it is positive in higher latitudes, thus contributing directly to polar warming amplification. However, the total radiative forcing change due to fire aerosol emissions would also include the aerosol indirect and surface albedo effects, and uncertainties remain concerning the sign of the total effect at high latitudes (*52*, *53*). In addition, among the modeling community, there is a large uncertainty with respect to the sign and magnitude of the contribution from wildfire-released aerosols to the direct aerosol effect (*51*). This uncertainty stems from this contribution being the sum of a large positive and negative term, from black carbon and organic aerosols, respectively. Furthermore, it is common practice to use overly smoothed biomass burning aerosol forcing to analyze radiation sensitivity to aerosols, but such smoothing has been shown to bias assessments of aerosol climatic impacts (*16*–*18*). In contrast, using fully coupled fire-aerosol modeling, as done here, avoids impacts from such input pre-processing on the estimation of fire contribution to the direct aerosol effect. The −0.05 W m^−2^ change that we find corresponds to 19.8% of the preindustrial global mean direct fire aerosol effect and −0.02% of the total net shortwave TOA budget. Purely in terms of global mean radiative forcing, such a negative feedback has an effect equivalent to a reduction of 0.91 ± 0.01% in atmospheric CO_2_ concentration [following the procedure of Myhre *et al.* (*62*)].

At the regional scale, we find that monthly burned area distributions are gamma distributed, as well as fire emissions. In addition, the shape parameters of the distributions are insensitive to the CO_2_ forcing, as demonstrated by the proportionality between mean and SD in different climate background states found across the IPCC reference regions (Fig. 7B). We link this finding to simple principles of statistical mechanics and argue that modeled fire activity can be approximated as a growth-and-decline process with stochastic fluctuations. The universal validity of this simple approximation across regions opens the door to the application of a reduced order fire model that combines both a deterministic and a stochastic component, which is a systematic strategy for many climate modeling aspects (*45*). In particular, a simple calibration of the shape and scale parameters to ecosystem and CO_2_ concentration, respectively, would be a straightforward implementation of a dynamic fire model in simulations of paleoclimate and of evolutionary human and animal habitat suitability (*63*, *64*).

More generally, our study offers a quantitative assessment of interactions between climate, lightning, and wildfires. The complexity of their relationships poses challenges in disentangling the roles of numerous compensating and amplifying factors that coexist in the real world. In this context, Earth System models remain invaluable to understand the sensitivity of such interactions to nonstationary forcings, such as the idealized CO_2_ concentration increase investigated here. However, models are not without their limitations. Multi-model intercomparisons help to quantify and understand some components of model-related uncertainty and have enabled great model improvements over the past decade (*31*, *51*). Nevertheless, calibration and validation remain crucial to lightning, fire, and aerosol modeling. Expanding the coverage, extending the time periods, and improving the reliability of observations for these challenging-to-measure variables should continue to be key scientific priorities.

## MATERIALS AND METHODS

### The CESM2

All our simulations are performed with the CESM2 (*34*). We run CESM2 with all atmosphere, land, sea-ice, and ocean models active. The lightning and fire models are detailed below, but we mention here some other model components important to this study. The atmosphere model is the Community Atmosphere Model version 6 (CAM6), and the land model is the Community Land Model version 5 (CLM5) (*34*, *65*). Both components use the same 1.9° × 2.5° horizontal grid and are coupled at a 30-min frequency, and there are 32 vertical levels in the atmosphere. Aerosols are simulated with the Modal Aerosol Model version 4 (MAM4), which describes four modes of aerosols with distinct microphysical and radiative properties (*38*). MAM4 simulates internal mixing within individual modes, and external mixing between modes, and it also includes consideration of aerosol aging and hydrophilic properties. Cloud microphysics are described by the Morrison-Gettelman scheme, which includes representation of cloud drop and crystal size distributions, as well as dependence of ice nucleation on aerosols (*66*). Last, of importance to our simulations under forced CO_2_ concentration changes, we note that CESM2 shows a high equilibrium climate sensitivity of +5.2 K, which is 1.5 K higher than the sixth Coupled Model Intercomparison Project (CMIP6) multi-model mean (*67*).

### Lightning model

We use the extreme learning machine regression tree lightning model (ELM-tree), implemented online within CAM6 (*35*). ELM-tree has been calibrated to a >25-year period of satellite-based lightning measurements and climate reanalysis data. It uses a regression tree approach, i.e., splitting the input space of climatic conditions in seven separate regimes, for which lightning rates are simulated with specific single hidden layer neural networks. ELM-tree predicts lightning rates only from large-scale climatic variables and is therefore suitable for use in Earth System models, such as CESM2. When compared to other lightning parameterizations, ELM-tree was shown to bring strong improvements in reproducing observed spatiotemporal lightning variability at the daily, seasonal, and interannual timescales (*35*). In this study, the lightning flash rates computed from the ELM-tree are directly passed to the fire model to serve as ignition source. Using such an online coupled lightning model allows representing the inherent relationships between availability of ignition sources and fire weather. Note that ELM-tree uses only climatic variables, and not aerosols due to the limitations in using aerosols for the development of a data-driven lightning model [see (*35*) for a detailed discussion].

### Fire model

We use the fire model implementation of Li *et al.* (*36*, *37*) in CLM5 (*65*). This model contains four components: agricultural fires in cropland, deforestation fires in tropical closed forests, peat fires in all regions, and all other fires (*37*). All components depend both on natural conditions through biomass availability or combustibility and on anthropogenic effects through fire initiation or suppression. Only the component “all other fires” is sensitive to lightning ignitions. We briefly summarize the fire model here, with an emphasis on the influence from ignitions, which is key to our coupled lightning-wildfire simulations. We refer to the original model publications for more model details (*36*, *37*).

Burned area from “agricultural fires in cropland” is calculated as

$$A_{b}=a_{1}f_{b}f_{\text{se}}f_{\text{ssn}}f_{\text{cr}}A_{g}$$ (5)

where $A_{b}$ is the burned area per time step and $A_{g}$ is the grid cell area. The socioeconomic factor $f_{\text{se}}$ depends on population density and gross domestic product (GDP). The seasonal factor $f_{\text{ssn}}$ depends on timing with respect to harvest and planting and accounts for rain. The cropland fraction of the grid cell is $f_{\text{cr}}$, and $a_{1}$ is a tuning parameter. Last, $f_{b}$ is the biomass factor, expressed as a piecewise function

$$f_{b}=\left\{\begin{array}{cc} 0 & \text{if}\;B_{\text{ag}}<B_{\text{low}}\\ \frac{B_{\text{ag}}-B_{\text{low}}}{B_{\text{up}}-B_{\text{low}}} & \text{if}\;B_{\text{low}}\leq B_{\text{ag}}\leq B_{\text{up}}\\ 1 & \text{if}\;B_{\text{ag}}>B_{\text{up}} \end{array}\right.$$ (6)

where $B_{\text{ag}}$ is the total aboveground biomass, and $B_{\text{up}}$ and $B_{\text{low}}$ are tuning parameters.

Burned area from “peat fires in all regions” is calculated as follows

$$A_{b}=a_{2}f_{c,p}f_{p}\left(1-f_{\text{sat}}\right)A_{g}$$ (7)

where $f_{p}$ is the peatland fraction in the grid cell, $f_{\text{sat}}$ is the fraction of the grid cell with the water table reaching the surface, and $a_{2}$ is a tuning parameter. The climatic factor $f_{c,p}$ is a function of the last 60-day precipitation in non-boreal peatlands and of upper-soil water content and temperature in boreal peatlands.

The “deforestation fires in tropical closed forests” depend on changes in prescribed land-use data. In this study, we keep fixed land-use forcing, and this component of the fire model can be ignored as a consequence.

The total fire count for all other fires per grid cell and per time step is calculated as

$$N_{f}=N_{\text{ig}}f_{b}f_{m}\left(1-f_{\text{hs}}\right)$$ (8)

where the biomass factor $f_{b}$ follows Eq. 6, and the human suppression factor $f_{\text{hs}}$ is a function increasing with population density. The combustibility factor $f_{m}$ is given by

$$f_{m}=\left\{\begin{array}{cc} f_{\text{RH}}\;f_{\beta } & \text{if}\;T_{s}>273.15\;K\\ 0 & \text{if}\;T_{s}\leq 273.15\;K \end{array}\right.$$ (9)

$$\begin{array}{c} f_{\text{RH}}=\left(1-w_{\text{RH}}\right)\left\{1-\text{max}\left[0,\text{min}\left(1,\frac{{\text{RH}}_{t}-{\text{RH}}_{\text{low}}}{{\text{RH}}_{\text{up}}-{\text{RH}}_{\text{low}}}\right)\right]\right\}+\\ w_{\text{RH}}\left\{1-\text{max}\left[0.75,\text{min}\left(1,\frac{{\text{RH}}_{30d}}{a_{3}}\right)\right]\right\} \end{array}$$ (10)

$$w_{\text{RH}}=1-\text{max}\left[0,\text{min}\left(1,\frac{B_{\text{ag}}-a_{4}}{a_{5}}\right)\right]$$ (11)

$$f_{\beta }=\left\{\begin{array}{cc} 1 & \text{if}\;\beta <\beta_{\text{low}}\\ \frac{\beta_{\text{up}}-\beta }{\beta_{\text{up}}-\beta_{\text{low}}} & \text{if}\;\beta_{\text{low}}\leq \beta \leq \beta_{\text{up}}\\ 0 & \text{if}\;\beta >\beta_{\text{up}} \end{array}\right.$$ (12)

where *T*_s_ is the surface air temperature; RH_*t*_ is the relative humidity at the current time step; RH_30d_ is the relative humidity averaged over the previous 30 days; β is the root zone wetness; and *a*_3_, *a*_4_, *a*_5_, *a*_6_, RH_low_, RH_up_, β_low_, and β_up_ are tuning parameters. The number of ignitions *N*_ig_ adds the contributions from anthropogenic (*I*_a_) and lightning (*I*_L_) ignition sources

$$N_{\text{ig}}=\left(I_{L}+I_{a}\right)A_{g}$$ (13)

where *I*_a_ is a function increasing with population density. To approximate cloud-to-ground flash ratio and lightning flash ignition efficiency, CLM5 scales the lightning flash rate density (*L*), computed from the ELM-tree, by a latitude-dependent factor to calculate *I*_L_ (*68*)

$$I_{L}=\frac{c_{1}}{c_{2}+c_{3}\text{cos}\;\left[3\;\text{min}\;\left(60,\varphi \right)\right]}\times L$$ (14)

where φ is the latitude and *c*_1_, *c*_2_, and *c*_3_ are fixed constants. Note that other climate-dependent parameterizations of the cloud-to-ground flash ratio exist [e.g., (*69*)], but we use the parameterization of Eq. 14 as now implemented in CLM5 (*36*, *68*). To compute the burned area, the total fire count is further multiplied by the fire average spread area, which is itself a function of wind speed, biomass, relative humidity, root zone wetness, as well as the human-related variables of population density and GDP. In the fire model, the influence of varying lightning *L* on burned area is therefore modulated by climatic, vegetation, and human variables, thus simulating the simultaneous effects of ignitions and fire weather. Note that we use the notion of burned area as the sum of the contributions from agricultural, peat, and all other fires.

Emissions of wildfire-released carbon are calculated from the burned area, as well as from the carbon density and combustion completeness factors of the burned biomass, which differ between leaves, stems, roots, and litter and depend on plant functional type as well (*36*). Furthermore, aerosol emissions are scaled to the carbon emissions by an emission factor specific to each combination of aerosol species and plant functional type (*70*).

### Simulation configurations

#### Spin-up

All transient simulations performed in this study are initialized from a preindustrial spin-up run, for which preindustrial external forcings are prescribed, such as greenhouse gas concentrations, aerosol emissions other than from biomass burning, solar activity, population density, and land use. This preindustrial spin-up run is initialized from the CESM2 preindustrial control run (*34*) that was performed for the CMIP6. Although our spin-up run starts from the equilibrium climate of the CMIP6 CESM2 preindustrial control run, it requires additional model simulation time to reach a stable quasi-equilibrium state. This is due mostly to recent modifications to CLM5 affecting the land vegetation and, to a smaller extent, to the interactive climate-lightning-wildfire setup, which was not used in the CMIP6 CESM2 preindustrial control run; instead, the latter run used a prescribed biomass burning aerosol forcing. In contrast, our preindustrial spin-up run uses the interactive climate-lightning-wildfire setup. With the objective to reach a quasi-equilibrium in the mean climate state, we perform the spin-up run over 345 years, at which point we assess the model convergence using different diagnostics (see the Supplementary Materials).

#### Transient simulations

We perform a set of four different transient simulations of 140 years, all branching from the final year of the spin-up run. Except for the modifications detailed below, all forcings and model configurations remain identical to those in the spin-up run. The transient simulations only differ from the spin-up and between themselves by the configuration of the climate-lightning coupling or by the CO_2_ forcing applied.

The first transient simulation is a preindustrial lightning-on run. Here, lightning-on means that the lightning scheme still follows the ELM-tree model, implemented online in the atmospheric component of CESM2. Preindustrial means that the CO_2_ forcing remains fixed at the 1850 level [284.7 parts per million (ppm)]. As such, this lightning-on preindustrial run is simply the continuation of the spin-up run for 140 more years, serving as a baseline of comparison for the other transient simulations.

The second transient simulation is a preindustrial lightning-clim run. In this model configuration, we turn off the lightning scheme and, instead, use the annual lightning climatology from the past 75 years of the spin-up run. The lightning climatology is computed from the monthly output of the spin-up run. To minimize artificial differences caused by the absence of sub-monthly and sub-daily variability in the monthly output used to create this climatology, we scale the spin-up run monthly climatology to the 2-hourly climatology from the LIS lightning data (*21*). In this manner, the total monthly lightning at each grid cell is taken from the spin-up run climatology, but the proportion of the monthly lightning occurring every 2 hours is determined by the LIS data. We acknowledge that the 2-hourly variability from the online ELM-tree lightning may be different to that from the LIS data. To verify potential impacts of this difference, we performed a short test run with the spin-up run monthly lightning climatology without refinement to 2-hourly variability; we found only negligible differences in simulated fires. Therefore, we estimate that differences between the ELM-tree and the LIS data in 2-hourly variability would cause even smaller differences in model output. By applying such a prescribed lightning climatology, there is an intentional decoupling of the fire weather and the lightning ignition sources that force the fire model. In this preindustrial lightning-clim run, we also keep the CO_2_ forcing fixed at the 1850 level (284.7 ppm).

The third transient simulation is a 1% CO_2_ lightning-on run. Lightning is still calculated online by the ELM-tree model. However, here, CO_2_ concentrations are increased by 1% per year, resulting in a quadrupling of CO_2_ concentrations (4 × CO_2_) at the end of the 140 model transient years. However, we emphasize that this simulation only captures the transient response of the climate system to +1% CO_2_ year^−1^ forcing, and it does not represent the climate at equilibrium with a fixed 4 × CO_2_ forcing.

Last, the fourth transient simulation is a 1% CO_2_ lightning-clim run. This simulation applies the +1% CO_2_ year^−1^ forcing, identical to the 1% CO_2_ lightning-on run. The lightning forcing is prescribed as the spin-up run lightning climatology, identical to the lightning-clim preindustrial run. As such, in addition to decoupling lightning and fire weather, this simulation does not represent any potential regional or global lightning changes in response to increased global temperatures.

Some important points concerning the forcing of all four transient simulations need to be reminded. First, anthropogenic impacts on wildfires are large and widespread, both in the real world (*1*) and in the fire model used here (*36*, *37*). Here, we keep the population density, the GDP, and the land use classes fixed to their 1850 levels. In the shared socioeconomic pathway scenarios (*71*), such factors change in time, affecting modeled fire changes (*31*). Thus, our 1% CO_2_ runs isolate the response of wildfires to changes in lightning, fire weather, and biomass under a CO_2_-driven global warming forcing, without any impacts from socioeconomic changes. Second, non-biomass aerosol emissions are also fixed at their 1850 levels, with the exception of dust emissions, which are calculated prognostically by CESM2. Last, the atmospheric CO_2_ concentrations are diagnostic, i.e., prescribed according to the forcing scenario. Thus, CO_2_ changes due to total biomass changes, burning vegetation, or changing ocean biochemistry are not accounted for in the radiative forcing. As such, we control the exact differences in greenhouse gas radiative forcing between the transient runs despite their differences in simulated wildfire emissions and magnitude of the CO_2_ fertilization effect.

### Comparisons and statistical significance

To compare climatic values between the different transient runs, we consistently use samples of the past 40 years of simulation. This is chosen as a compromise between reaching the 4 × CO_2_ concentration and analyzing a sample of size large enough to account for interannual variability. We note here that, over the past 40 years of simulation, the CO_2_ concentration increase in the 1% CO_2_ runs ranges between 2.7 and 4 times the preindustrial conditions (284.7 ppm); again, we emphasize that the climate response is transient, and not in equilibrium with such CO_2_ levels.

When performing multiple hypothesis testing (i.e., at multiple model grid cells), we report statistical significance by controlling for an FDR of 5% (α_FDR_ = 0.05). The FDR approach adjusts for test multiplicity by placing a strict limit on the fraction of significant grid cell results that are spurious (*72*). In this procedure, we derive the critical *P* value for a test being considered significant ($P_{\text{FDR}}^{*}$) as follows

$$P_{\text{FDR}}^{*}=\underset{i=1\ldots N}{\text{max}}\left(P_{i}:P_{i}\leq \frac{1}{N}\alpha_{\text{FDR}}\right)$$ (15)

where *N* is the number of local hypothesis tests, and the $P_{i}$ are the local test *P* values sorted in ascending order ($P_{1}<P_{2}<\ldots <P_{N}$). This method ensures that $\alpha_{\text{FDR}}$ is the upper limit for the overall expected proportion of erroneously rejected local null hypotheses among the rejections, i.e., the FDR. This expectation holds regardless of the unknown proportion of local tests having true null hypotheses. In contrast, reporting significance on a local test by local test basis only controls the probability of each individual true null hypothesis being erroneously rejected. As such, there is no overall control, and the proportion of erroneously rejected null hypotheses is an unknown function of the proportion of null hypotheses that should be rejected. Because the rejected hypothesis tests are of interest, e.g., nonzero change or nonzero correlation, it is preferable to control the proportion of those rejections that are meaningful. We refer to Wilks (*72*) for details and examples. When comparing two samples of annual mean values, we perform two-tailed *t* tests. When reporting correlations, we use the Pearson correlation coefficient and use the *t* statistic to test that it is significantly different from zero. When reporting significance of a multiple linear regression, we perform *F* tests to evaluate the significance of the collective set of predictors.

### Fire aerosol radiative forcing calculations

We investigate the impact of wildfire-released aerosols on the surface radiative budget by linking fire emissions, AOD, and fluxes of shortwave radiation. To isolate the wildfire-related changes in AOD, we remove the dust- and sea salt–related components of AOD and denote the resulting quantity AOD*. Note that all aerosol sources other than from dust, sea salts, and wildfires use prescribed emissions, which are identical in all simulations. As such, differences in AOD* (Fig. 8A) are directly attributable to wildfire-released aerosol load differences.

Following established and recommended procedures from the climate community (*49*), we focus on the radiative forcing contribution from the direct fire aerosol effect [$F^{\left(d\right)}$] to the net shortwave flux at the TOA. We analyze this effect in both all-sky and clear-sky conditions. We use the diagnostic radiation calculation capabilities of CESM2. In particular, during the simulations, the radiation routine is also performed assuming the absence of all fire-emitted aerosols. These diagnostic calculations do not influence the simulations and only generate the hypothetical no–fire-aerosol radiative fluxes as outputs. We consider downward fluxes as positive and define

$$\left\{\begin{array}{c} F_{\text{all},f}^{\left(d\right)}=F_{\text{all}}-F_{\text{all},\text{no}-f}\\ F_{\text{clear},f}^{\left(d\right)}=F_{\text{clear}}-F_{\text{clear},\text{no}-f} \end{array}\right.$$ (16)

where all and clear denote in all-sky and clear-sky conditions, respectively, no-f denotes the no-fire-aerosols fluxes, and f emphasizes that the resulting fluxes account only for the effect from fire aerosols. The *F* terms without superscripts denote the net TOA shortwave fluxes. Equation 16 provides an unbiased estimate of the direct fire aerosol effect because it accounts for absorbing aerosols above clouds and does not overestimate cooling from scattering aerosols below clouds [see (*49*) for detailed explanations]. We use Eq. 16 in the following decomposition

$$F_{\text{all},f}^{\left(d\right)}=F_{\text{clear},f}^{\left(d\right)}+F_{\text{res},f}^{\left(d\right)}$$ (17)

where the residual $F_{\text{res},f}^{\left(d\right)}$ denotes the additional direct fire aerosol effect when conditions deviate from clear-sky conditions. As such, $F_{\text{res},f}^{\left(d\right)}$ results mostly from highly nonlinear processes involving clouds, fire aerosols, and their combined influence on the radiation budget. We emphasize that all $F_{\cdot ,f}^{\left(d\right)}$ terms account for the direct aerosol effect from fire aerosols only. Because scattering from fire aerosols generally dominates absorption, $F_{\text{all},f}^{\left(d\right)}$ and $F_{\text{clear},f}^{\left(d\right)}$ are typically negative, i.e., fire aerosols reflect solar radiation out. But because the role of aerosols is more prominent in clear-sky conditions, $F_{\text{clear},f}^{\left(d\right)}$ is typically more negative than $F_{\text{all},f}^{\left(d\right)}$. That is, one would expect $F_{\text{res},f}^{\left(d\right)}$ to be positive in most areas: The contribution of fire aerosols to the total reflection of shortwave radiation out of the atmosphere is smaller in the presence of clouds.

Under this framework, we compare the response of all three terms of Eq. 17 to the 1% CO_2_ year^−1^ forcing. We define

$$\left\{\begin{array}{c} \Delta \left[F_{\text{all},f}^{\left(d\right)}\right]=F_{\text{all},f,{\text{CO}}_{2}}^{\left(d\right.}-F_{\text{all},f,\text{pi}}^{\left(d\right)}\\ \Delta \left[F_{\text{clear},f}^{\left(d\right)}\right]=F_{\text{clear},f,{\text{CO}}_{2}}^{\left(d\right)}-F_{\text{clear},f,\text{pi}}^{\left(d\right)}\\ \Delta \left[F_{\text{res},f}^{\left(d\right)}\right]=F_{\text{res},f,{\text{CO}}_{2}}^{\left(d\right)}-F_{\text{res},f,\text{pi}}^{\left(d\right)} \end{array}\right.$$ (18)

where the subscripts pi and CO_2_ denote the preindustrial and 1% CO_2_ runs, respectively.

We note here that it is common to diagnose not only the direct aerosol effect but also the indirect and surface albedo effects by using equations similar to Eq. 16 (*12*, *49*). In particular, the indirect, i.e., associated with cloud changes, and surface albedo effects could be computed with the no–fire-aerosol radiative fluxes only. However, such methods assume simulations with different aerosol emissions but a same climate state. In our case, the climate change in the 1% CO_2_ runs causes other effects, not related to fire aerosols, that influence the radiative budget. The changes in the indirect and surface effects from fire aerosols can, therefore, not be easily separated from these other influences, as can be illustrated by two simple examples. First, one can expect the change in fire aerosol burden to affect cloud properties. But these properties are also affected by, e.g., changes in dust and sea salt aerosols, in atmospheric humidity, and in temperature profiles. Second, changes in fire aerosol deposition do affect albedo of snow- and ice-covered areas, but albedo change in such areas is dominated by the reduction of their extent caused by global warming. For both of these examples, subtracting the diagnostic no–fire-aerosol radiative fluxes does not isolate the fire-aerosol–induced component from the ones induced by changes in climate, dust, and/or sea salts. As such, estimating the radiative forcing from the indirect aerosol effect and from the aerosol-induced surface albedo change attributable specifically to wildfire-released aerosols would require questionable assumptions, and we prefer to use the simple but unambiguous formulation of Eq. 17.
