# Supplementary material for: Quantifying CO2 forcing effects on lightning, wildfires, and climate interactions
Source: Sci Adv. 2025 Feb 12;11(7):eadt5088. doi: 10.1126/sciadv.adt5088 (PMC11817939; doi:10.1126/sciadv.adt5088)
Supplement: Supplementary file 1 — Supplementary Text Figs. S1 to S17 Table S1 [file sciadv.adt5088_sm.pdf]

Science Advances
AAAS

Supplementary Materials for

Quantifying CO_2 forcing effects on lightning, wildfires, and climate interactions

Vincent Verjans *et al.*

Corresponding author: Vincent Verjans, vincent.verjans@bsc.es;
Christian L. E. Franzke, christian.franzke@pusan.ac.kr

Sci. Adv. **11**, eadt5088 (2025)
DOI: 10.1126/sciadv.adt5088

This PDF file includes:

Supplementary Text
Figs. S1 to S17
Table S1

Supplementary Text

Mean climate-lightning-wildfire equilibrium state

After 345 years of spin-up run under 1850 level forcings, the mean climate state is close to equilibrium. We apply the non-parametric Mann-Kendall trend test to a range of global-scale variables (*74, 75*). For example, we find no significant trend over the last 75 years of the spin-up in global mean 2m temperature, mean precipitation, total burned area, total lightning flash rate, and total sea ice extent ($p > 0.25$). The trend in global total vegetation carbon stocks is still statistically significant ($p < 0.05$), but the linear trend is 0.01% yr^{-1}, much reduced compared to 0.14% yr^{-1} in the first 75 years. Global maps for changes in different variables during the spin-up run are shown in fig. S1.

Figure S3 focuses on burned area and lightning over the last 75 years of the spin-up run. The annual mean global burned area is $(7.82 \pm 0.51) \times 10^6$ km^2 yr^{-1}, where ± denotes 1 standard deviation (σ) of the inter-annual variability. This compares well with the 1997-2021 estimate of $(7.90 \pm 0.66) \times 10^6$ km^2 yr^{-1} from the Global Fire Emission Database 5 (GFED5) (*73*). As shown in fig. S3G, the time series show a good correspondence for long-term mean, inter-annual variability, and monthly cycle. Comparing the spatial patterns of modeled and observed burned area (fig. S3 A,B), we find a realistic distribution of fires in our spin-up run; the global spatial correlation with observations is $r = 0.50$. It is important to remind here that the model uses climate forcing, as well as socioeconomical variables and land use fixed to the pre-industrial levels. Thus, by design, it is not capable of capturing spatio-temporal patterns caused by human-related fires over the observational period. This is most evident in the tropical forests, where modeled burned area is very low (fig. S3A) because tropical deforestation fires are turned off. Another example is the low-degree of fire suppression in regions such as western North America and the Mediterranean. Since such effects impact the burned area distribution, we find that the correlation statistic $r = 0.50$ compares satisfactorily with a recent model inter-comparison (*58*), which accounted for human-related fire impacts and present-day climate forcing. The model inter-comparison mean $\pm 1\sigma$ range for global spatial correlation is $r = 0.54 \pm 0.15$ (*58*). Finally, we have computed the monthly climatology of both the spin-up run burned area and the GFED5 data set at all grid cells (fig. S3E). We find that 32.6% of the grid cells have a statistically significant correlation between the modeled and GFED5

monthly climatologies (p satisfies a false discovery rate $\alpha_{FDR} = 0.05$, see Materials and Methods). In addition, comparing fig. S3E and fig. S3B, it is clear that the significant correlations are mostly achieved in areas with high observed burned area, except for tropical forests where deforestation fires are not simulated.

Concerning lightning, the spin-up run yields an annual mean global flash (fl.) rate of 40.9±0.9 fl. s^{-1}. This is within observational uncertainties of the estimated global total of 44.0 with uncertainty range ±5.0 fl. s^{-1} (*19, 76*). This is achieved without an artificial lightning scaling factor commonly applied in lightning modeling studies [e.g., (*24, 27, 77*)]. However, comparing results with the LIS 2002-2013 time series covering only the ±38° latitude domain, we find an underestimation of the modeled lightning rate (33.9 ± 0.8 fl. s^{-1}) with respect to the LIS observations (41.8 ± 0.6 fl. s^{-1}) in this latitude band (orange dashed limits in fig. S3 C,D,F). The inter-annual and monthly variability of model and observations are nevertheless in good agreement (1σ of 0.8 versus 0.6 fl. s^{-1}, and monthly peak-to-peak of 11.4 versus 7.7 fl. s^{-1}, respectively, see also fig. S3H). Comparing the spatial distribution with the combined data from the OTD and LIS missions [1995-2013 (*21, 76*)], our spin-up run reproduces important lightning features, such as the land-to-ocean contrast, the peaks in the tropical continental regions, as well as elevated lightning in the southeastern United States, Australia, and eastern Asia (fig. S3 C,D). The global spatial correlation coefficient is $r = 0.78$ (fig. S3 C,D). Comparing the modeled and observed monthly climatologies (fig. S3F), we find a statistically significant correlation for 73% of all grid cells, and 83% over land (p satisfies $\alpha_{FDR} = 0.05$, two-sided t-test). Here also, we note that the modeled lightning rates are obtained under simulated 1850 climate, while the observations are taken over the period 1995-2013, which affects the comparison at the regional and global scales.

Derivation of the steady-state solution to the growth-and-decline stochastic differential equation

In this section, we provide the mathematical derivation of the steady-state probability distribution given in Section *Universality of the wildfire response at the regional scale*. We denote $x(t)$ the monthly regional burned area, x^* the equilibrium state for $x(t)$, τ the characteristic timescale for return to x^*, and ξ the strength of burned area fluctuations. We write a simple growth-and-decline

stochastic differential equation (same as Eq. (2)):

$$\frac{dx}{dt} = \frac{x^* - x}{\tau} + \sqrt{2\xi x}\,\eta(t) \tag{S1}$$

where $\eta(t)$ represents Gaussian uncorrelated noise. This equation has an associated Fokker-Planck equation for the probability density function, $p(x,t)$ [e.g., (*78*)]:

$$\frac{\partial p(x,t)}{\partial t} = -\frac{\partial}{\partial x}\left[\left(\frac{x^* - x}{\tau}\right)p(x,t)\right] + \frac{1}{2}\frac{\partial^2}{\partial x^2}\left[2\xi x p(x,t)\right] \tag{S2}$$

which simplifies to (same as Eq. (3)):

$$\frac{\partial p(x,t)}{\partial t} = -\frac{\partial}{\partial x}\left[\left(\frac{x^* - x}{\tau}\right)p(x,t)\right] + \xi\frac{\partial^2}{\partial x^2}\left[x p(x,t)\right] \tag{S3}$$

We write $p_{ss}(x)$ as the steady-state solution, corresponding to:

$$\frac{\partial p_{ss}(x)}{\partial t} = 0 \tag{S4}$$

and we substitute in Eq. (S3):

$$\begin{aligned} 0 &= -\frac{\partial}{\partial x}\left[\left(\frac{x^* - x}{\tau}\right)p_{ss}(x)\right] + \xi\frac{\partial^2}{\partial x^2}\left[x p_{ss}(x)\right] \\ \left(\frac{x^* - x}{\tau}\right)p_{ss}(x) &= \xi\frac{\partial}{\partial x}\left[x p_{ss}(x)\right] \\ \frac{1}{\xi\tau}\left(\frac{x^*}{x} - 1\right) &= \frac{1}{x p_{ss}(x)}\frac{\partial}{\partial x}\left[x p_{ss}(x)\right] \end{aligned} \tag{S5}$$

Letting $q(x) = x p_{ss}(x)$:

$$\frac{1}{q(x)}\frac{\partial q(x)}{\partial x} = \frac{1}{\xi\tau}\left(\frac{x^*}{x} - 1\right) \tag{S6}$$

Integrating, noting that x, $p_{ss}(x)$, and $q(x)$ are all strictly positive, and using c_0 as constant of integration:

$$\begin{aligned} \log[q(x)] &= \frac{1}{\xi\tau}\int\left(\frac{x^*}{x} - 1\right)dx \\ \log[q(x)] &= \frac{1}{\xi\tau}\left[x^*\log(x) - x\right] + c_0 \\ q(x) &= \exp(c_0)\,\exp\left[\frac{x^*}{\xi\tau}\log(x)\right]\,\exp\left(\frac{-x}{\xi\tau}\right) \\ q(x) &= \exp(c_0)\,x^{\frac{x^*}{\xi\tau}}\,\exp\left(\frac{-x}{\xi\tau}\right) \end{aligned} \tag{S7}$$

Replacing $q(x)$ by $xp_{ss}(x)$:

$$p_{ss}(x) = \exp(c_0)\, x^{\frac{x^*}{\xi\tau}-1} \exp\left(\frac{-x}{\xi\tau}\right) \tag{S8}$$

Finally, since $p_{ss}(x)$ is a probability density function, the left-hand-side must integrate to 1, which allows to find the correct expression of the constant term $\exp(c_0)$:

$$p_{ss}(x) = \frac{1}{\mathbf{\Gamma}\left(\frac{x^*}{\xi\tau}\right)(\xi\tau)^{\frac{x^*}{\xi\tau}}}\, x^{\frac{x^*}{\xi\tau}-1} \exp\left(\frac{-x}{\xi\tau}\right) \tag{S9}$$

where boldface $\mathbf{\Gamma}$ denotes the gamma function. Equation (S9) is the gamma probability density function written in Eq. (4):

$$p_{ss}(x) \sim \Gamma\left(\frac{x^*}{\xi\tau}, \xi\tau\right) \tag{S10}$$

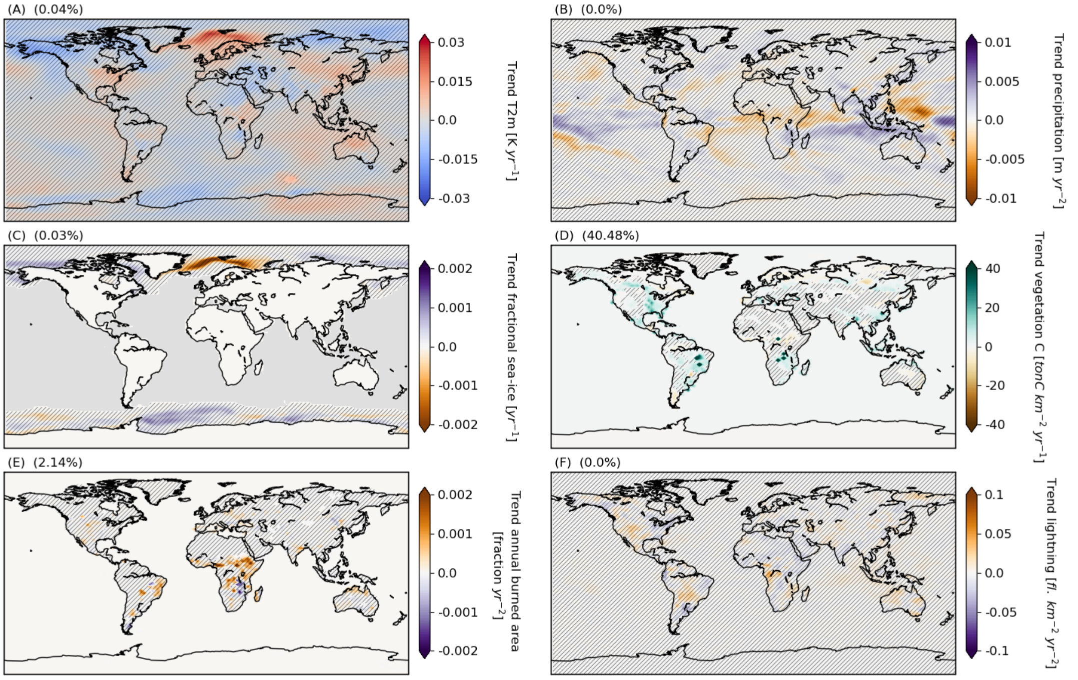

Figure S1: **Trends in spin-up run.** Linear trends over the last 75 years of the spin-up run, shown for (A) 2m temperature, (B) total precipitation, (C) fractional sea ice cover, (D) total vegetation, (E) fractional total burned area, (F) lightning flash rate density. Statistical significance in a Mann-Kendall trend test (*74, 75*) is reported by controlling for a false discovery rate $\alpha_{FDR} = 0.05$ (see Materials and Methods). Areas where the trend is not significant are hatched. For each variable, the percentage of grid cell with statistically significant trend is reported on top of the sub-panel. Relevant grid cells are defined as follows. For sea ice, at least one time step has non-zero sea ice cover. For vegetation and burned area, all land grid cells out of Antarctica. For 2m temperature, total precipitation, and lightning, all grid cells are considered relevant.

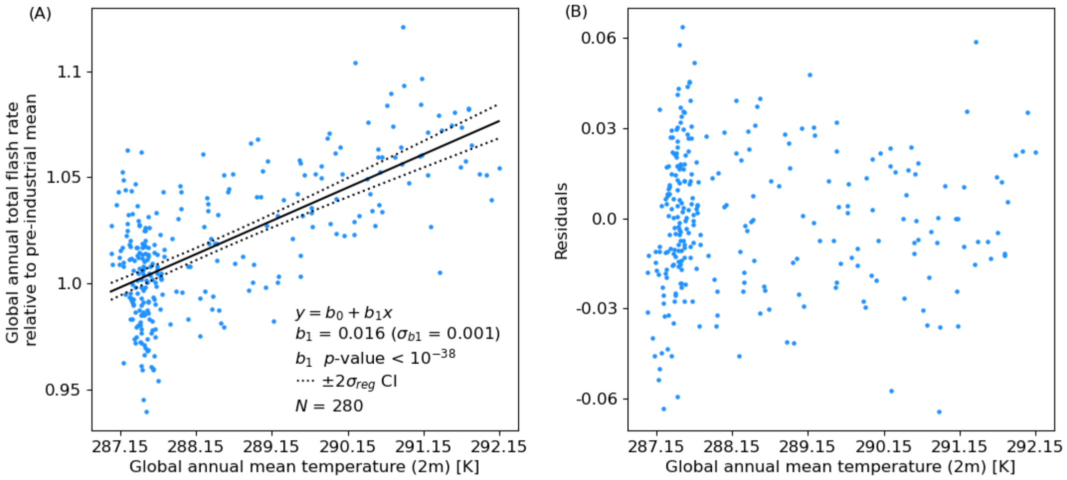

Figure S2: **Linear temperature - lightning global relation.** (A) All global total annual mean lightning flash rate values, relative to the pre-industrial mean, as a function of the corresponding global annual mean 2m temperature. The solid black line shows the linear fit, with equation, linear coefficient, linear coefficient standard error (σ_{b1}), linear coefficient p-value, and number of data points (N) given. The dotted lines show the $\pm 2\sigma_{reg}$ confidence interval, where σ_{reg} is the standard error in the estimated regression line. In (B), the residuals from the linear fit show no discernible structure, supporting the null hypothesis of a linear sensitivity. Note that all 140 years from the lightning-on versions of the pre-industrial and 1% CO_2 runs are shown.

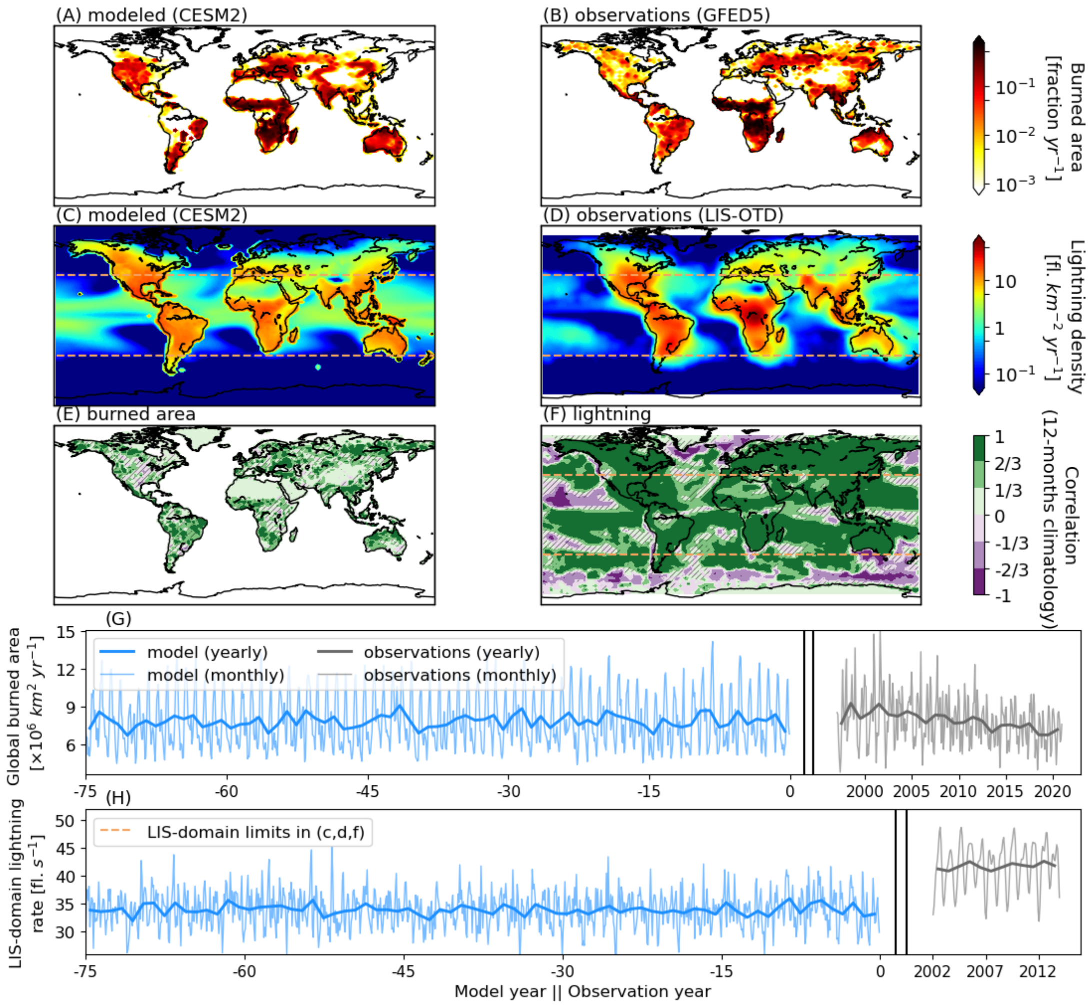

Figure S3: **Equilibrium fire and lightning regimes.** Top-row: mean burned area from (A) the last 75 years of the spin-up run and (B) the 1997-2021 data product GFED5 (*73*). Second row: mean lightning flash rate density from (C) the last 75 years of the spin-up run and (D) the 1995-2013 combined LIS-OTD data product (*21, 76*). Third row: correlation in monthly climatology between the modeled and observational (E) burned area and (F) lightning products. Time series show the (g) global total burned area and (h) total lightning flash rate within the LIS domain limits (dashed lines in C,D,F). In (E,F), hatching denotes correlation not significant, evaluated with a two-tailed t-test by controlling for $\alpha_{FDR} = 0.05$. Note logarithmic color scales in (A,B,C,D).

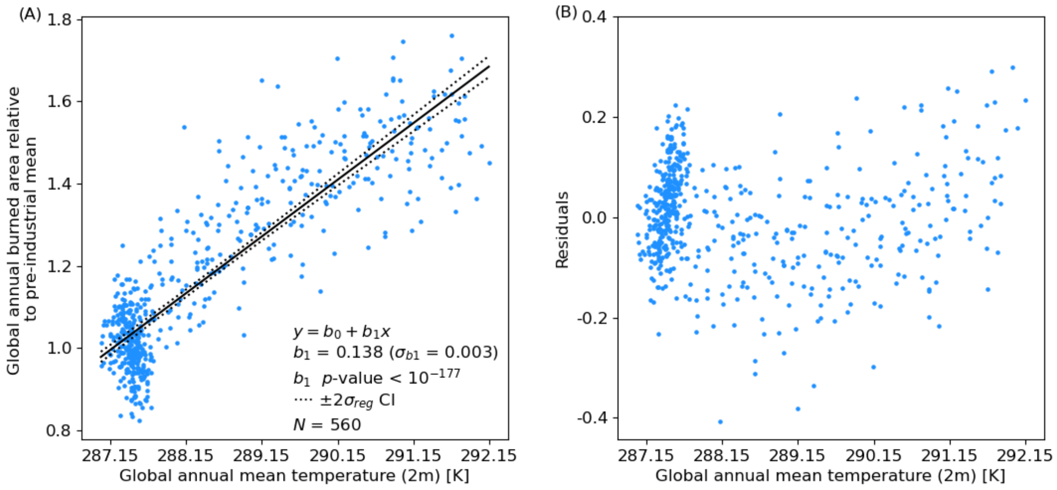

Figure S4: **Linear temperature - burned area global relation.** (A) All global total annual mean burned area values, relative to the pre-industrial mean, as a function of the corresponding global annual mean 2m temperature. The black line shows the linear fit, with equation, linear coefficient, linear coefficient standard error (σ_{b1}), linear coefficient p-value, and number of data points (N) given. The dotted lines show the $\pm 2\sigma_{reg}$ confidence interval, where σ_{reg} is the standard error in the estimated regression line. In (B), the residuals from the linear fit show no discernible structure, supporting the null hypothesis of a linear sensitivity. Note that all 140 years from all simulations (pre-industrial lightning-on and lightning-clim, and 1% CO_2 lightning-on and lightning-clim) have been pooled to increase the sample size.

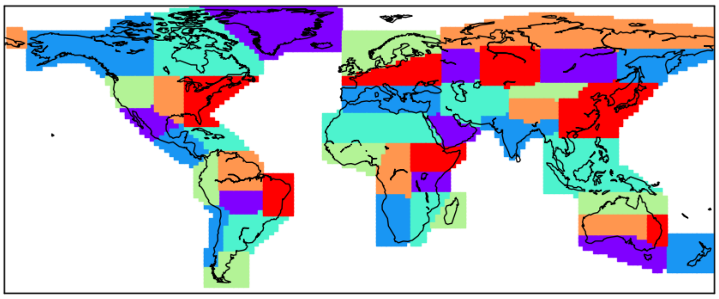

Figure S5: **Region delineations.** Reference regions from the Intergovernmental Panel on Climate Change (*43*). Since regions are used to aggregate burned area, only land-based regions, and excluding Antarctica, are shown.

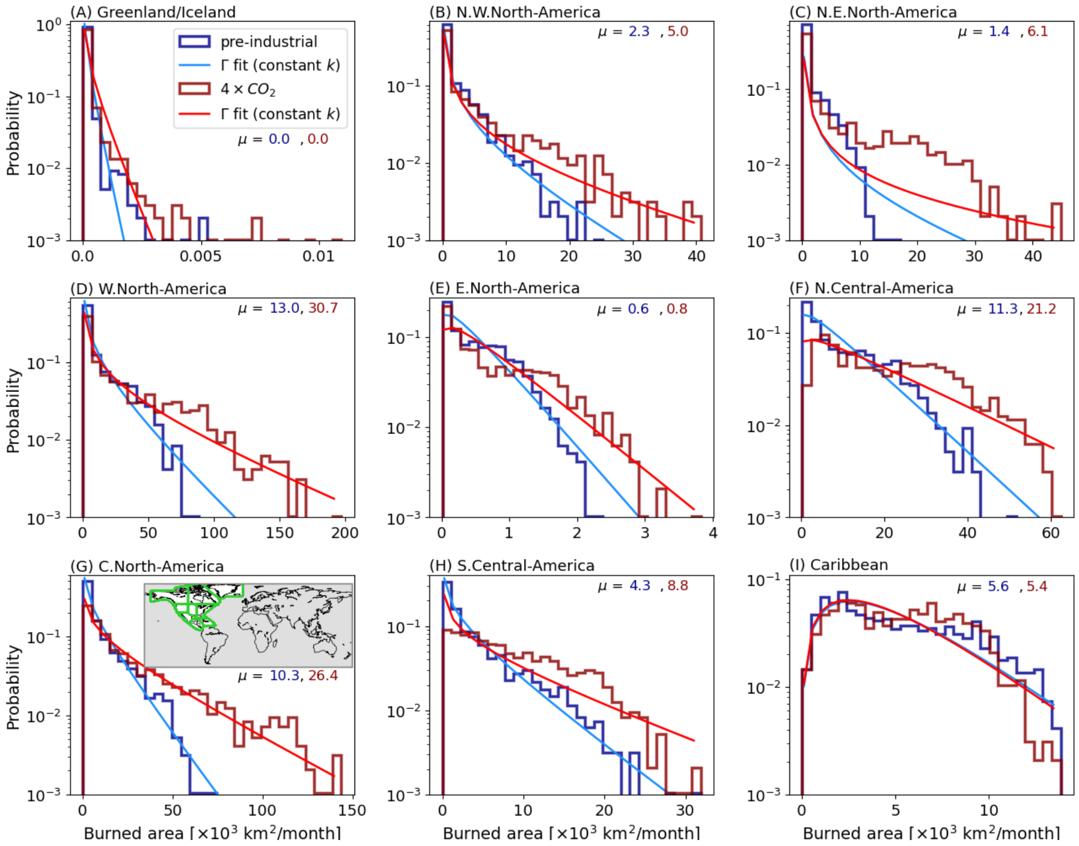

Figure S6: **Regional-scale burned area distributions.** Same as Fig. 6 for 9 IPCC regions. Histograms of regional total monthly burned area over the last 40 years of simulation of the (dark blue) pre-industrial runs, and (dark red) 1% CO_2 runs. Probabilities on the y-axes are shown with a logarithmic scale. Sub-panels (A to I) show different regions, with name given on top of each sub-panel, and delineations shown in the inset map in (G). See Fig. 6 caption for all details.

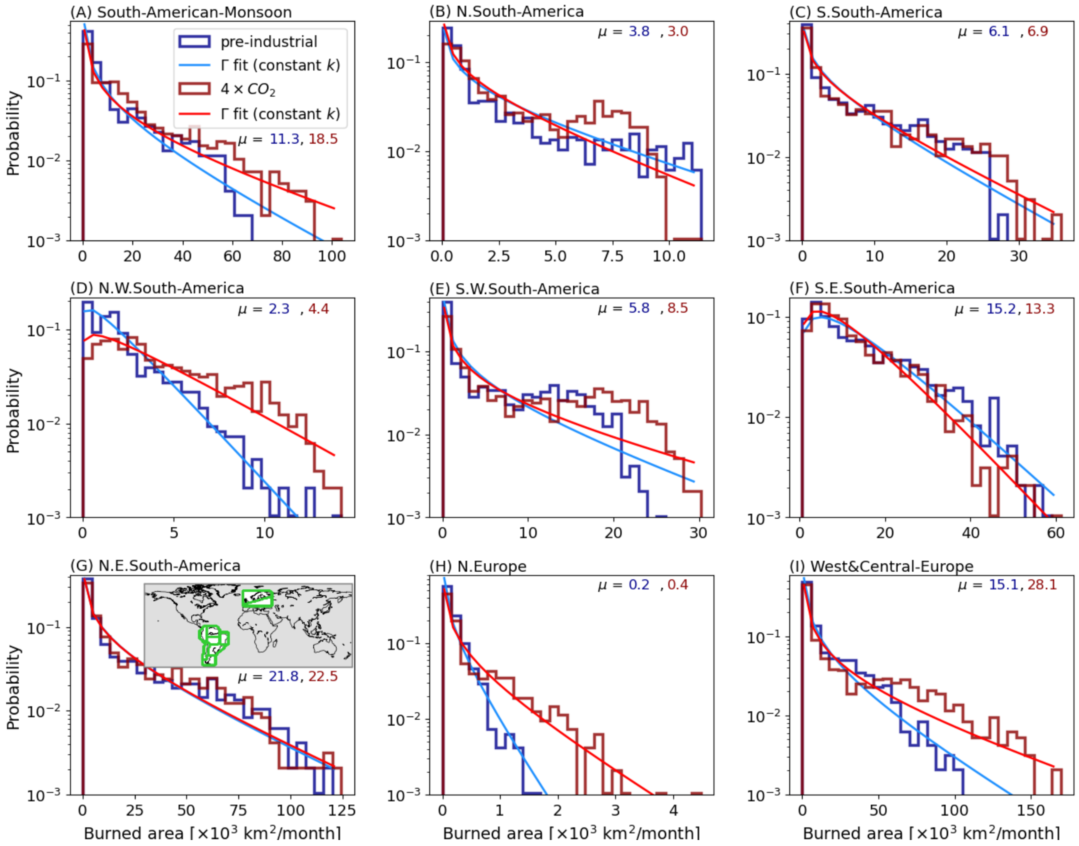

Figure S7: **Regional-scale burned area distributions.** Same as Fig. 6 for 9 IPCC regions. Histograms of regional total monthly burned area over the last 40 years of simulation of the (dark blue) pre-industrial runs, and (dark red) 1% CO_2 runs. Probabilities on the y-axes are shown with a logarithmic scale. Sub-panels (A to I) show different regions, with name given on top of each sub-panel, and delineations shown in the inset map in (G). See Fig. 6 caption for all details.

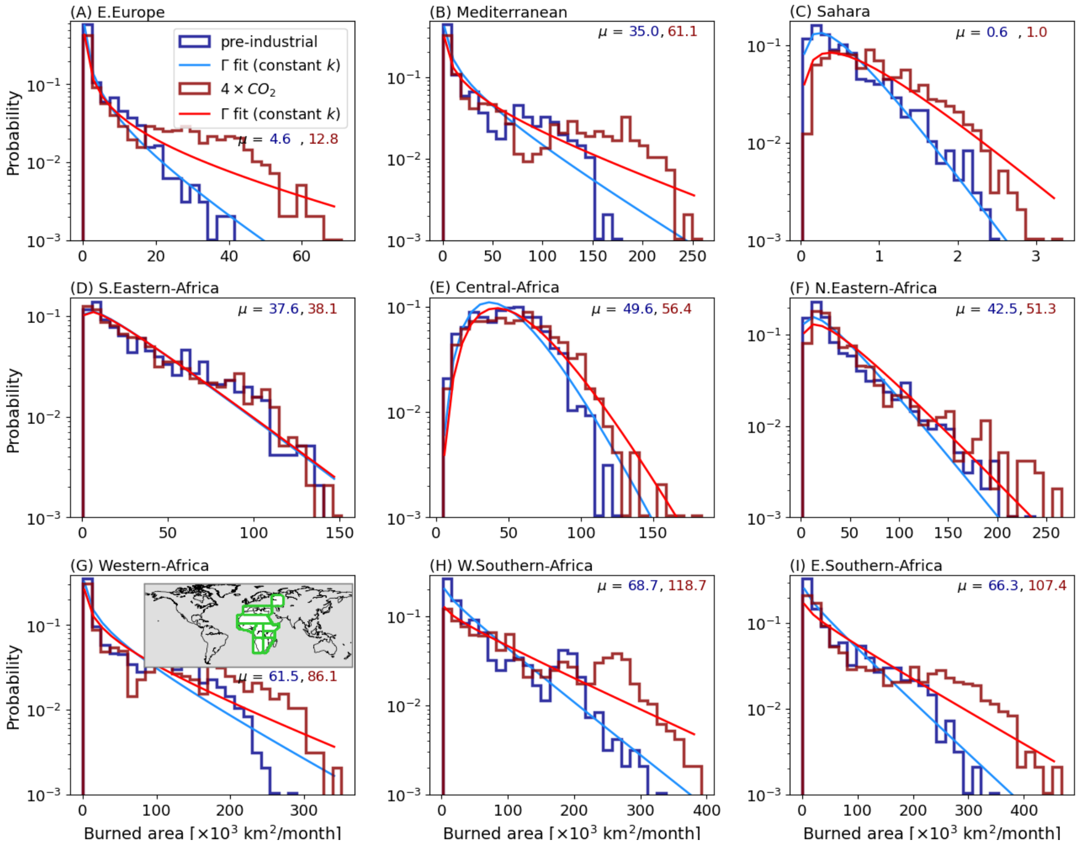

Figure S8: **Regional-scale burned area distributions.** Same as Fig. 6 for 9 IPCC regions. Histograms of regional total monthly burned area over the last 40 years of simulation of the (dark blue) pre-industrial runs, and (dark red) 1% CO_2 runs. Probabilities on the y-axes are shown with a logarithmic scale. Sub-panels (A to I) show different regions, with name given on top of each sub-panel, and delineations shown in the inset map in (G). See Fig. 6 caption for all details.

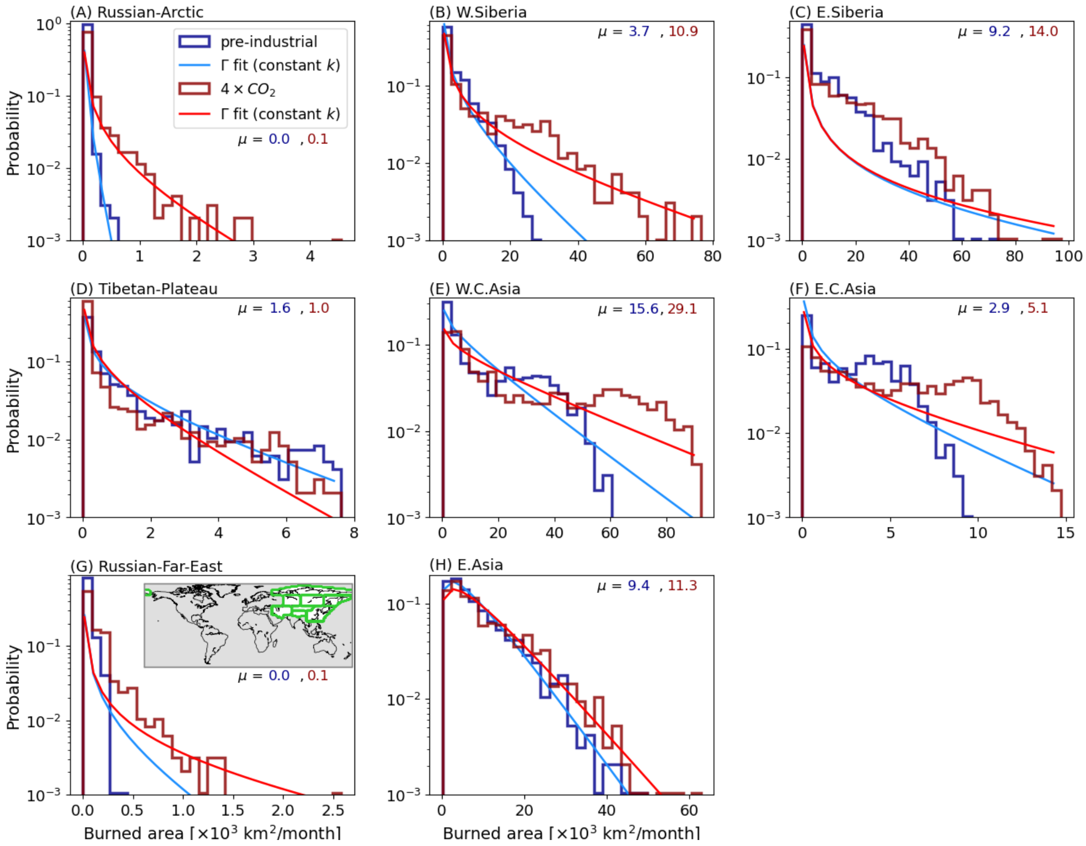

Figure S9: **Regional-scale burned area distributions.** Same as Fig. 6 for 9 IPCC regions. Histograms of regional total monthly burned area over the last 40 years of simulation of the (dark blue) pre-industrial runs, and (dark red) 1% CO_2 runs. Probabilities on the y-axes are shown with a logarithmic scale. Sub-panels (A to I) show different regions, with name given on top of each sub-panel, and delineations shown in the inset map in (G). See Fig. 6 caption for all details.

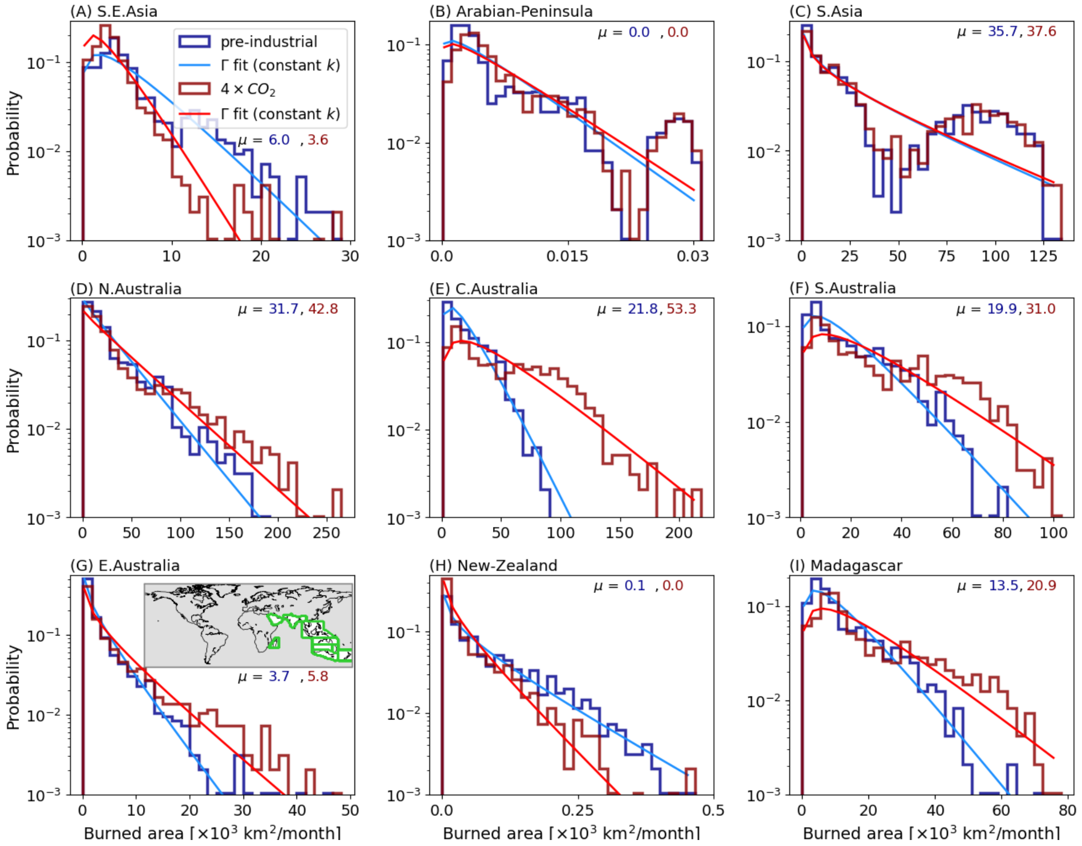

Figure S10: **Regional-scale burned area distributions.** Same as Fig. 6 for 9 IPCC regions. Histograms of regional total monthly burned area over the last 40 years of simulation of the (dark blue) pre-industrial runs, and (dark red) 1% CO_2 runs. Probabilities on the y-axes are shown with a logarithmic scale. Sub-panels (A to I) show different regions, with name given on top of each sub-panel, and delineations shown in the inset map in (G). See Fig. 6 caption for all details.

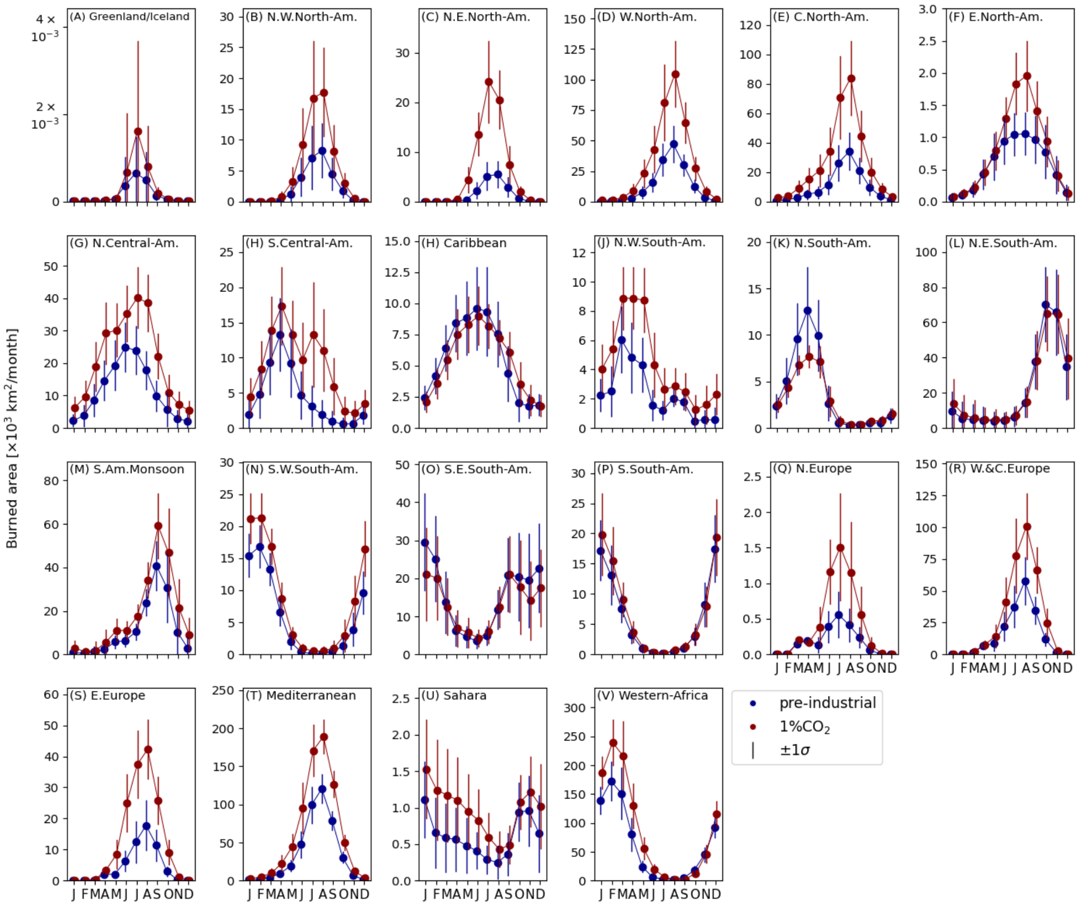

Figure S11: **Burned area monthly climatology.** Regional total burned area monthly climatology calculated over the last 40 years of simulation of the (dark blue) pre-industrial runs and (dark red) 1% CO_2 runs. Vertical ranges show ±1 standard deviation (±1σ). Sub-panels (A to V) show 22 IPCC reference regions, and the other 22 regions are shown in fig. S12. Region name is given in each sub-panel, and delineations are shown in fig. S5.

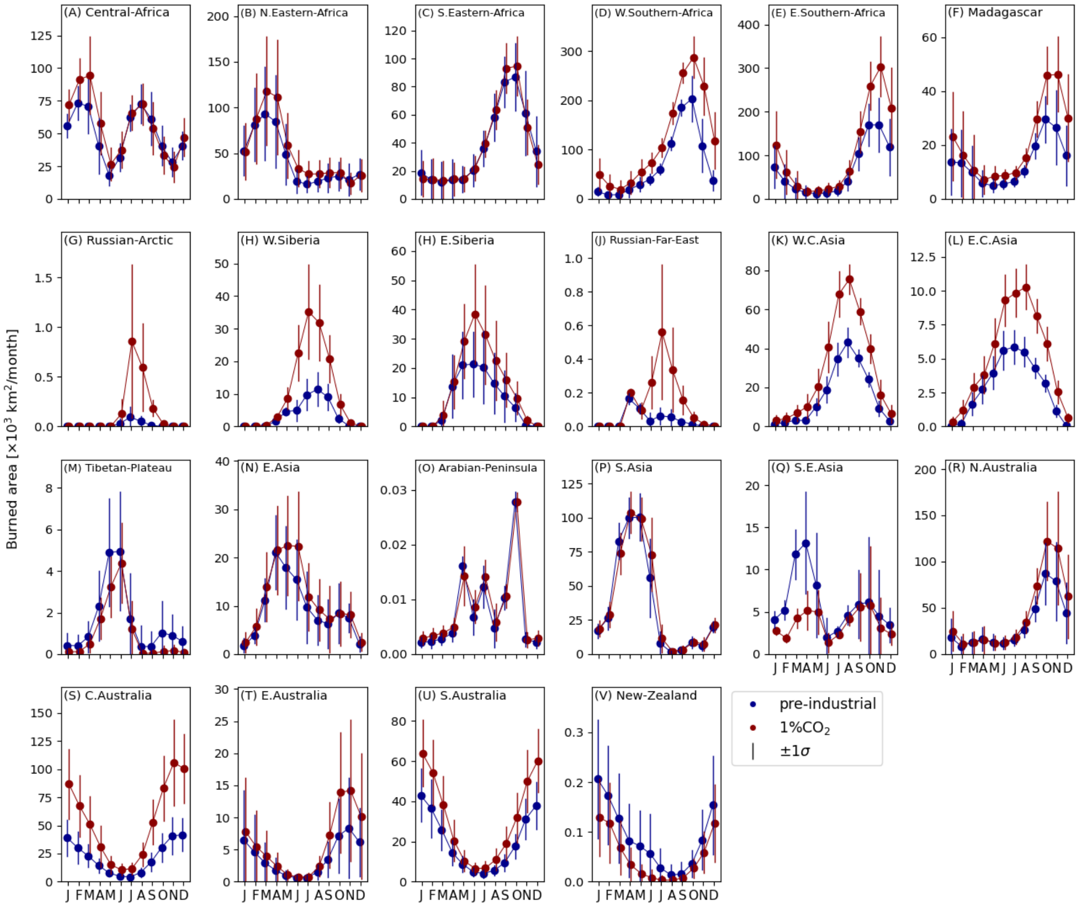

Figure S12: **Burned area monthly climatology.** Regional total burned area monthly climatology calculated over the last 40 years of simulation of the (dark blue) pre-industrial runs and (dark red) 1% CO_2 runs. Vertical ranges show ±1 standard deviation (±1σ). Sub-panels (A to V) show 22 IPCC reference regions, and the other 22 regions are shown in fig. S11. Region name is given in each sub-panel, and delineations are shown in fig. S5.

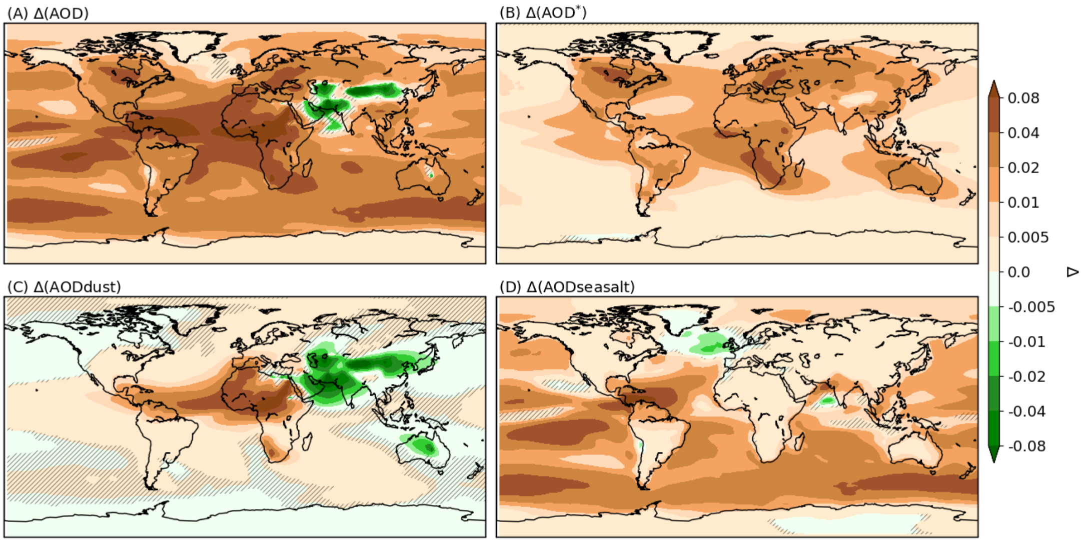

Figure S13: **Changes in aerosol optical depth (AOD) and its components.** Annual mean difference averaged over the last 40 years of simulation between the 1% CO_2 and pre-industrial runs for (A) the total AOD (Δ(AOD)), and the components of Δ(AOD) due to (B) fire aerosols (Δ(AOD*)), (C) dust (Δ(AODdust)), and (D) sea salts (Δ(AODseasalt)). Note that the lightning-on and lightning-clim runs have been pooled to increase the sample size. Note also that AOD and its components are taken at 550 nm, and all are dimensionless. Note also the non-linear color scaling. Hatching denotes annual mean difference not significant, evaluated with a two-tailed t-test by controlling for $\alpha_{FDR} = 0.05$.

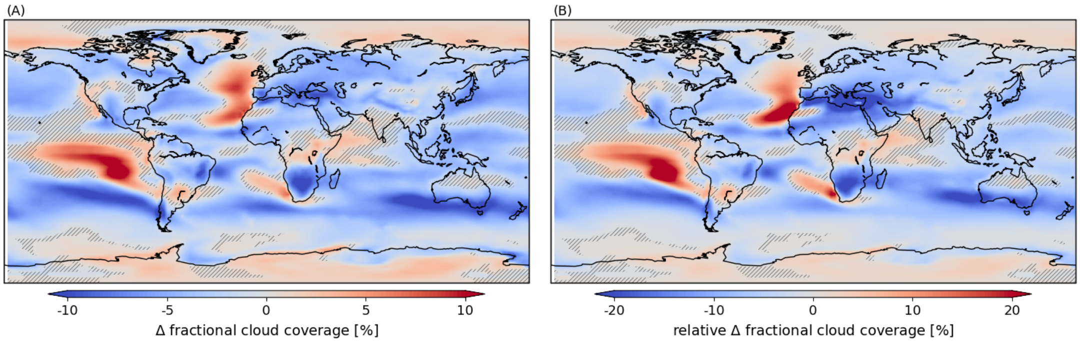

Figure S14: **Cloud cover changes.** Annual mean difference averaged over the last 40 years of simulation between the 1% CO_2 and pre-industrial runs for fractional cloud coverage in (A) absolute terms, and (B) relative to the pre-industrial run values. Note that the lightning-on and lightning-clim runs have been pooled to increase the sample size. Hatching denotes annual mean difference not significant, evaluated with a two-tailed t-test by controlling for $\alpha_{FDR} = 0.05$.

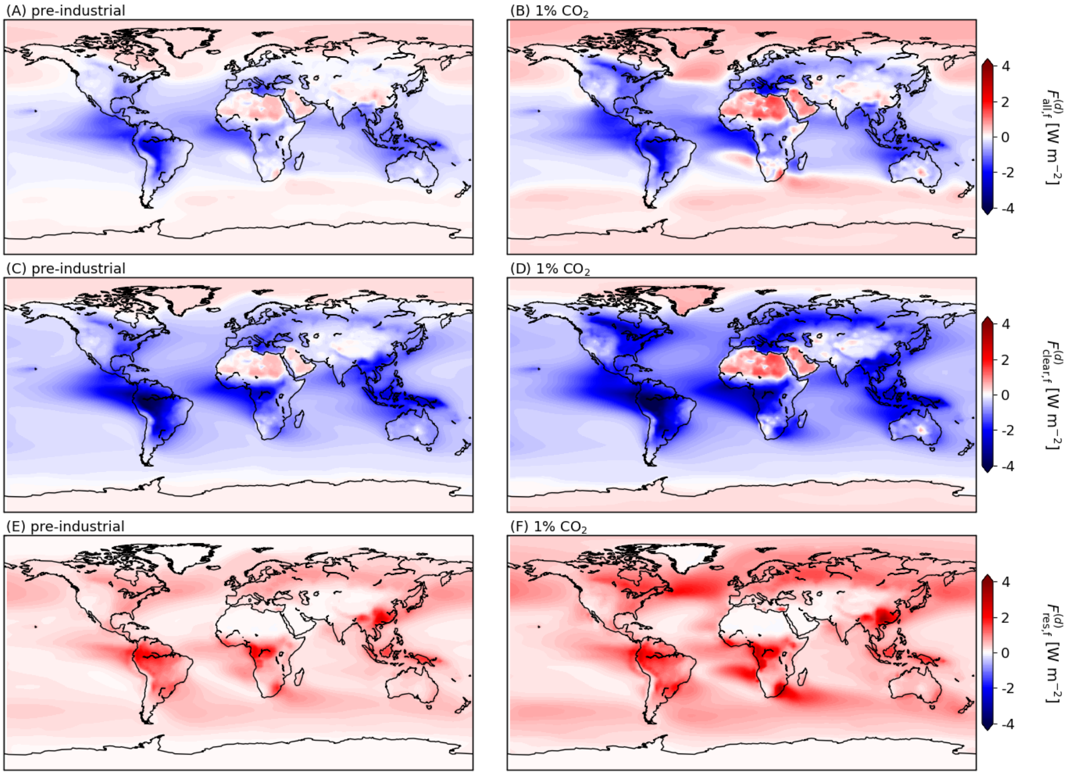

Figure S15: **Radiative forcing through direct fire aerosol effect.** Maps of direct fire aerosol effect on shortwave radiative forcing averaged over the last 40 years of simulation for the (A,C,E) pre-industrial runs and (B,D,F) 1% CO_2 runs. Fluxes (positive downward) are (A,B) the all-sky radiative forcing from the direct fire aerosol effect ($F^{(d)}_{\text{all,f}}$), (C,D) the clear-sky radiative forcing from the direct fire aerosol effect ($F^{(d)}_{\text{clear,f}}$), and (E,F) the difference $F^{(d)}_{\text{res,f}} = F^{(d)}_{\text{all,f}} - F^{(d)}_{\text{clear,f}}$. See Materials and Methods for details on the flux calculations. Note that the lightning-on and lightning-clim runs have been pooled to increase the sample size.

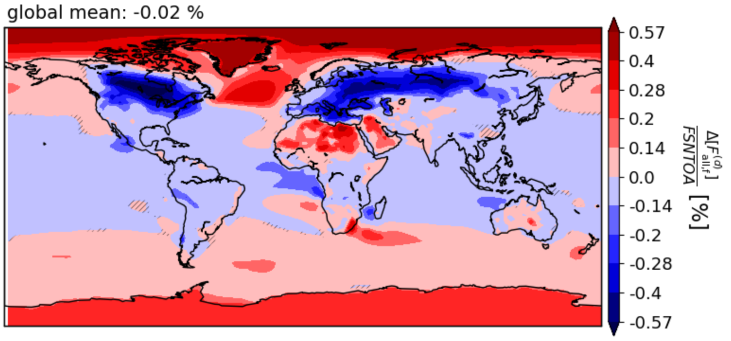

Figure S16: **Change in direct fire aerosol effect relative to total shortwave radiation.** Annual mean difference averaged over the last 40 years of simulation between the 1% CO_2 and pre-industrial runs for the all-sky radiative forcing from the direct fire aerosol effect ($F^{(d)}_{\mathrm{all,f}}$), which is shown relative to the pre-industrial run mean net shortwave flux at the top-of-atmosphere (*FSNTOA*). The global area-weighted mean value is given on top of the figure. Note that the lightning-on and lightning-clim runs have been pooled to increase the sample size. Note also the non-linear color scaling. Hatching denotes annual mean difference not significant, evaluated with a two-tailed t-test by controlling for a false discovery rate $\alpha_{FDR} = 0.05$

.

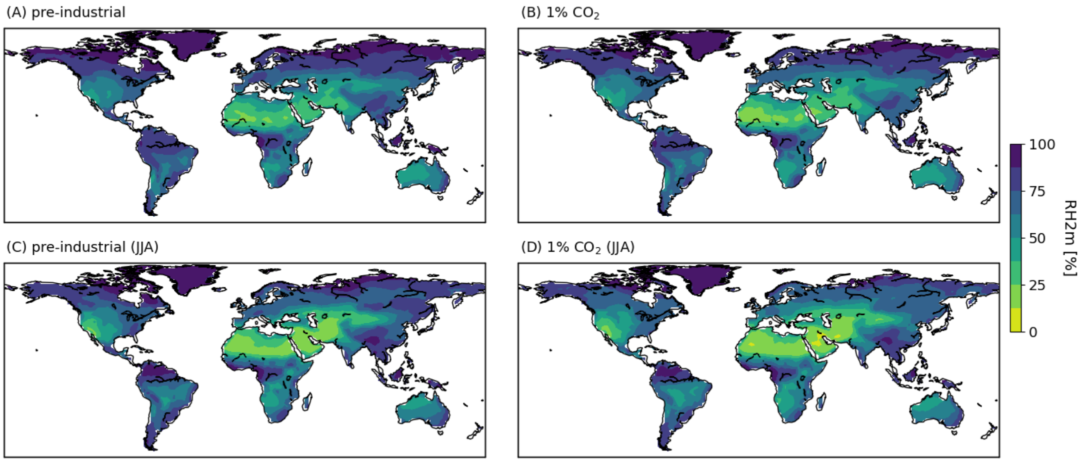

Figure S17: **2m relative humidity.** Relative humidity at 2m height (RH2m), averaged over the last 40 years of simulation. Top-row: annual mean values in the (A) pre-industrial runs, and (B) 1% CO_2 runs. Bottom-row: June-July-August (JJA) mean values in the (C) pre-industrial runs, and (D) 1% CO_2 runs. Note that the lightning-on and lightning-clim runs have been pooled to increase the sample size.

Table S1: **CO_2-forced changes in statistics of monthly fire activity.** (next page) Monthly total burned area per IPCC reference region. For each region, we calculate the mean (μ) and coefficient of variation (CV) under the pre-industrial (subscript *pi*) and 1% CO_2 (subscript CO_2) climate scenarios, as well as the percentile (π) change for events of given percentile-level in the pre-industrial to the 1% CO_2 climate scenario. For example, $\pi_{CO_2}(0.5_{pi})$ denotes percentile-level in 1% CO_2 climate scenario of the 0.5 percentile-level magnitude event in the pre-industrial climate. Distributions are computed only over the last 40 years of simulation, but pooling the lightning-on and lightning-clim runs to increase the sample size. Percentiles are calculated analytically from the fitted Γ distributions, shown in figs. S6-S10. Region delineations are shown in fig. S5.

[*] The Arctic is not an IPCC reference region, and is taken here as all grid cells North of 60°N.

[**] For the global values, μ and CV are calculated from the global total values. However, we do not fit a Γ distribution to the global total values, and percentile changes are calculated by weighting each region-specific percentile change by its mean burned area.

Region	μ_{pi} [10^3 km^2 month^{-1}]	μ_{CO_2} [10^3 km^2 month^{-1}]	CV_{pi}	CV_{CO_2}	$\pi_{CO_2}(0.5_{pi})$	$\pi_{CO_2}(0.95_{pi})$	$\pi_{CO_2}(0.99_{pi})$
Greenland/Iceland	0.00	0.00	2.69	3.04	0.38	0.84	0.94
N.W.North America	2.27	4.97	1.62	1.50	0.43	0.87	0.95
N.E.North America	1.41	6.07	1.68	1.48	0.45	0.87	0.94
W.North America	12.96	30.73	1.29	1.20	0.35	0.81	0.91
C.North America	10.31	26.41	1.20	1.11	0.29	0.74	0.87
E.North America	0.57	0.80	0.82	0.90	0.38	0.87	0.96
N.Central America	11.32	21.15	0.86	0.68	0.30	0.79	0.91
S.Central America	4.34	8.76	1.16	0.76	0.33	0.81	0.92
Caribbean	5.56	5.41	0.67	0.56	0.51	0.95	0.99
N.W.South America	2.34	4.44	0.94	0.73	0.29	0.78	0.90
N.South America	3.83	2.99	1.26	0.94	0.56	0.97	1.00
N.E.South America	21.79	22.55	1.24	1.17	0.49	0.95	0.99
South American Monsoon	11.29	18.45	1.28	1.10	0.42	0.89	0.96
S.W.South America	5.81	8.47	1.11	0.99	0.43	0.90	0.97
S.E.South America	15.24	13.26	0.81	0.79	0.56	0.97	1.00
S.South America	6.07	6.85	1.14	1.16	0.47	0.93	0.98
N.Europe	0.18	0.44	1.28	1.42	0.35	0.80	0.91
West&Central Europe	15.14	28.09	1.32	1.30	0.40	0.87	0.95
E.Europe	4.63	12.78	1.46	1.25	0.38	0.82	0.91
Mediterranean	35.03	61.09	1.20	1.10	0.38	0.86	0.95
Sahara	0.60	0.97	0.80	0.61	0.30	0.81	0.93
Western Africa	61.47	86.12	1.05	1.03	0.42	0.90	0.97
Central Africa	49.64	56.36	0.46	0.50	0.41	0.91	0.98
N.Eastern Africa	42.50	51.26	0.94	0.94	0.42	0.91	0.98
S.Eastern Africa	37.60	38.11	0.86	0.86	0.49	0.95	0.99
W.Southern Africa	68.74	118.67	1.01	0.82	0.34	0.83	0.93
E.Southern Africa	66.26	107.40	1.04	1.02	0.36	0.85	0.94
Madagascar	13.45	20.94	0.85	0.82	0.33	0.83	0.94
Russian Arctic	0.02	0.15	2.95	2.51	0.37	0.80	0.90
W.Siberia	3.66	10.86	1.31	1.30	0.37	0.80	0.90
E.Siberia	9.22	14.04	1.25	1.16	0.49	0.93	0.98
Russian Far East	0.04	0.14	1.46	1.59	0.46	0.89	0.95
W.C.Asia	15.63	29.14	0.97	0.91	0.33	0.81	0.92
E.C.Asia	2.86	5.10	0.80	0.74	0.38	0.86	0.95
Tibetan Plateau	1.56	0.98	1.43	1.67	0.60	0.99	1.00
E.Asia	9.36	11.32	0.87	0.87	0.42	0.91	0.98
Arabian Peninsula	0.01	0.01	0.99	0.89	0.47	0.94	0.99
S.Asia	35.7	37.57	1.08	1.03	0.49	0.94	0.99
S.E.Asia	5.97	3.63	0.88	0.87	0.72	0.99	1.00
N.Australia	31.74	42.80	1.05	1.12	0.40	0.89	0.97
C.Australia	21.81	53.33	0.80	0.77	0.20	0.64	0.81
E.Australia	3.71	5.82	1.40	1.33	0.38	0.87	0.95
S.Australia	19.92	31.02	0.81	0.77	0.33	0.83	0.94
New-Zealand	0.09	0.05	1.10	1.33	0.67	0.99	1.00
Arctic*	0.14	0.90	2.02	1.71	0.36	0.75	0.85
Global**	671.62	1009.51	0.28	0.28	0.39	0.86	0.95
